# Targeting PSMB5-induced PANoptosis in bladder cancer: multi-omics insights and TCM candidate discovery

**DOI:** 10.3389/fimmu.2025.1656682

**Published:** 2025-12-02

**Authors:** Zhe Chang, Jirong Wang, Jiajia Cao, Xinpeng Fan, Kunpeng Li, Chenyang Wang, Yalong Zhang, Li Wang, Jianwei Yang, Siyu Chen, Li Yang

**Affiliations:** 1 Department of Urology, Second Hospital of Lanzhou University, Lanzhou, China; 2 Gansu Province Clinical Research Center for Urinary System Disease, Lanzhou, China; 3 Department of Hematology, Second Hospital of Lanzhou University, Lanzhou, China

**Keywords:** bladder cancer, PANoptosis, machine learning, prognostic model, PSMB5, single-cell, TCM

## Abstract

**Background:**

Bladder cancer (BLCA) is among the most common malignancies worldwide, with significant mortality rates. The function of PANoptosis in BLCA, as a controlled process of programmed cell death, remains largely unelucidated. The study aimed to elucidate the role of PANoptosis-related genes in BLCA and investigate their molecular mechanisms, prognostic significance, and therapeutic potential.

**Methods:**

By analyzing differentially expressed genes in BLCA from The Cancer Genome Atlas (TCGA) and PANoptosis-associated genes, we discovered 98 genes associated with PANoptosis. Functional enrichment and consensus clustering identified molecular subtypes linked to these genes. A prognostic model was developed via LASSO regression based on these genes. Subsequent analyses assessed clinical significance, characteristics of the immunological milieu, and treatment responsiveness. Systematic screening with machine learning (ML) identified PSMB5 as a pivotal gene, with its functional importance further clarified using single-cell sequencing and Mendelian randomization analysis (MR). *In vitro* research confirmed the biological activities of PSMB5 in BLCA. Molecular docking demonstrated PSMB5’s binding affinity with traditional Chinese medicines (TCMs).

**Results:**

Clustering of 98 PANoptosis-associated genes revealed molecular subgroups A and B. A prognostic approach identified high-risk and low-risk cohorts, revealing considerable disparities in clinical characteristics and immunological landscapes across the groups. ML and MR identified PSMB5 as a risk factor in BLCA. Single-cell sequencing revealed that PSMB5 expression is predominantly associated with three cell lines linked to lymph node metastases. *In vitro* findings demonstrated that PSMB5 knockdown inhibited the proliferation and migration of BLCA cells while promoting apoptosis, whereas overexpression has the opposite effect. Molecular docking revealed a robust binding affinity between PSMB5 and five TCMs.

**Conclusions:**

A prognostic model incorporating PANoptosis-related genes was developed for stratifying BLCA risk and assessing the immune microenvironment. PSMB5 has been recognized as a crucial therapeutic target, exhibiting dual importance in the molecular etiology of BLCA and traditional Chinese medicine intervention.

## Introduction

Bladder cancer (BLCA), the tenth most common disease worldwide, presents a significant challenge to healthcare systems internationally due to its very high treatment costs per patient (1, 2). Despite a larger incidence rate in men, women generally experience poorer outcomes due to factors such as menstruation and cystitis (3, 4). Tobacco smoking and occupational exposures are unequivocally significant risk factors (5). The definitive method for diagnosing and monitoring BLCA, encompassing non-muscle-invasive BLCA (NMIBC) and muscle-invasive BLCA (MIBC), is invasive cystoscopy paired with pathological biopsy (6, 7). NMIBC typically necessitates transurethral resection of bladder tumor (TURBT). At the same time, radical cystectomy (RC) is employed for MIBC or NMIBC patients who do not react to bacillus Calmette-Guérin (BCG) therapy, as well as for tumors with the highest progression risk (8, 9). Nevertheless, this treatment is inaccessible to several patients, whereas RC significantly diminishes patient quality of life (10, 11). BLCA is a significant therapeutic challenge, requiring advanced research and innovative treatment strategies to improve prognosis and life quality (12).

Programmed cell death (PCD), considered a meticulously regulated type of cell death under normal settings, can impede the growth of neoplastic cells and maintain tissue homeostasis (13, 14). The three most thoroughly researched forms of PCD—pyroptosis, apoptosis, and necroptosis—interact during the PCD process rather than functioning independently of one another (15, 16). PANoptosis, a novel concept of programmed cell death presented by American researcher Malireddi et al., is induced by a complex PANoptosis that activates downstream molecules and all three programmed cell death pathways (17, 18). Moreover, four unique PANoptosome complexes have been structurally and functionally characterized at the molecular level, namely Z-DNA binding protein 1 (ZBP1) (19), absent in melanoma 2 (AIM2) (20), receptor-interacting protein kinase 1 (RIPK1) (21), and NOD-like receptor family, pyrin domain containing 12 (NLRP12) (22). These multiprotein platforms amalgamate elements from pyroptosis, apoptosis, and necroptosis pathways to orchestrate inflammatory cell death via PANoptosis (23). The characterization encompasses the identification of essential regulatory proteins, interaction networks, and activation mechanisms in response to pathogenic or cellular stress signals (24).

The relationship between PANoptosis and malignancies may yield novel insights into tumor initiation and development, as well as identify unique therapeutic targets and treatment strategies. Researchers synthesized ultrasmall Bi2Sn2O7 as an effective inducer of PANoptosis, consistently activating PANoptosis in hepatocellular carcinoma (25). The chlorin e6 photosensitizer generates reactive oxygen species, whereas Jolkinolide B specifically targets and activates the PANoptosis switch, thereby synergistically causing apoptosis in gastric cancer cells (26). Moreover, research indicates that baicalin mitigates disc degeneration, and licochalcone B reduces pulmonary fibrosis by regulating PANoptosis, underscoring PANoptosis as a pivotal mechanism in TCM (27, 28). Exploring the therapeutic potential of traditional Chinese medicine targeting PANoptosis-related genes is essential for cancer treatment.

Nonetheless, the mechanistic foundation and pathophysiological significance of PANoptosis in BLCA remain to be clarified, and no relevant TCM studies are focusing on genes associated with PANoptosis, particularly in BLCA. This research utilized datasets of PANoptosis and BLCA to categorize BLCA patients into two subgroups, examining immunological risk and checkpoints between these subtypes. Subsequently, we developed a predictive model for BLCA. We performed Mendelian Randomization (MR), single-cell sequencing, and many *in vitro* assays to further evaluate the biological function and molecular mechanism of the core gene PSMB5. The therapeutic potential of PSMB5 was investigated by reverse drug discovery and molecular docking.

## Methods

### Data collection about BLCA and PANoptosis

The data for BLCA patients was obtained from the TCGA and encompasses transcriptomic and clinical information. A total of 431 files (comprising 406 cases) were acquired, consisting of 412 tumor files and 19 normal files. To facilitate analysis, the TPM format was employed for the data. Owing to the incomplete clinical data, certain information was omitted from the clinical study. The 277 PANoptosis genes were discerned from the existing literature (29).

### Filtration of genes associated PANoptosis and BLCA

We computed the t-statistics, LogFC, and P value using the “eBays” function. The comparative limma analysis (version 4.3.3) identified 1.5-fold differently expressed transcripts (adj.P<0.05) in BLCA, indicating PANoptosis-related molecular signatures in the TCGA cohort (30). We intersected the two gene sets to produce a collection of PANoptosis-associated BLCA genes (BLCA-PANs) for subsequent investigation.

### Function enrichment analysis

Enrichment analysis for the BLCA-PANs was performed using the “org.Hs.eg.db” and “clusterProfiler” R packages (version 4.3.3) (31). All P values were less than 0.05. Based on protein-protein interaction (PPI) analysis, we identified communications and many key genes within the BLCA-PANs (STRING: functional protein association networks (string-db.org)).

### Unsupervised clustering and survival analysis

Unsupervised consensus clustering utilizing the K-means algorithm was executed with ConsensusClusterPlus to identify novel BLCA molecular subtypes based on characteristic gene profiles (32). The empirical cumulative distribution function (CDF) was utilized to ascertain the appropriate number of clusters (33). We subsequently evaluated prognostic variations using the R package “survival” and BLCA-PANs signatures in connection to clinical outcomes and immune infiltration.

### Foundation of prognosis model

We conducted univariate Cox regression analysis to identify genes with *P*-values of less than 0.05. Following data preprocessing, the raw data were randomly divided into training and testing sets (1:1 ratio) using the “caret” package. A prognostic model was developed using LASSO stepwise regression (34). Utilizing 10-fold cross-validation, the λ value associated with the smallest mean squared error and its standard error (SE) was identified as the stable solution, and regularization methods were employed to reduce the hazards of overfitting (35). This procedure discovered features with non-zero coefficients and produced coefficient path visualizations and cross-validation error curves. Hazard ratios (HR) and their 95% confidence intervals (CI) were derived using the model gene coefficients, with findings displayed in a forest plot format.

Compute the AUC (Area Under Curve) and the *P*-value for survival analysis. The threshold for the training set is established at *P* < 0.01, whereas the threshold for the test set is set at *P* < 0.05. The training set AUC exceeds 0.65, while the test set AUC surpasses 0.63 (36). Feature selection and model training are conducted solely on the training set, with the test set used only for final validation and verification. The formula for calculating the risk score is as follows: $RiskScore=\sum_{i=1}^{n}\;(\beta_{i}\times Expression_{i})$. Risk stratification thresholds are established by the predetermined median risk score, categorizing patients into high-risk and low-risk groups for a comparative survival study, including overall survival (OS) and progression free survival (PFS). Graph the C-index, AUC curve, and decision curve analysis (DCA) to assess the correlation between the model risk score and clinical baseline variations (37). Develop a nomogram utilizing clinical parameters and generate the calibration curve. The “maftools” R package was used to evaluate tumor mutational burden (TMB) (38).

### Somatic mutation and immune landscape analysis

We conducted several analyses based on risk stratification, encompassing RNA stemness score (RNAss), immunological subtypes, Gene Set Enrichment Analysis (GSEA), and Single Sample Gene Set Enrichment Analysis (ssGSEA). RNAss is a score system derived from transcriptome data that evaluates stem cell characteristics, primarily utilized to analyze the stemness aspects of cells in tumor or other tissue samples. 1000 permutation tests determined levels of significance (*P* < 0.05, FDR<0.25) to guarantee robust statistical inference (39, 40). Comparisons between high-risk and low-risk groups revealed immunophenotypic difference across four dimensions: effector cells, signaling pathways, functional annotations, and the repertoire of Immune checkpoint (IC) molecules. Immune cell infiltration analysis primarily relies on the CIBERSORTx algorithm. Furthermore, we computed Tumor Microenvironment (TME) scores derived from the stromal score, immune score, ESTIMATE score, and tumor purity to evaluate variations in the tumor microenvironment (41). Additionally, the IMvigor 210 dataset from the immunotherapy cohort was utilized for relevant assessment, encompassing Tumor Immune Dysfunction and Exclusion (TIDE) and Microsatellite Instability (MSI), which can elucidate immune evasion and immunotherapy for high-risk and low-risk individuals (42). Ultimately, we conducted a drug susceptibility prediction study using the “oncoPredict” R package, which is grounded in the prognostic model.

### Key feature gene screening and single gene correlation analysis

We utilized four machine learning techniques to identify significant feature genes for the model. Boruta does a top-down feature relevance analysis by systematically comparing the significance of characteristics with that of shadow attributes generated through the random permutation of the original qualities (43). It assesses significance by its permuted equivalents and systematically removes extraneous aspects to stabilize the evaluation. Support Vector Machine Recursive Feature Elimination (SVM-RFE) was applied to a dataset subjected to 10-fold cross-validation, with the number of folds set at 10. This produced indices for the training and testing sets. Following the application of the SVM-RFE algorithm to each training fold, features were prioritized according to their average rank (44). The Random Forest model is trained utilizing the “randomForest” package, with a specification of 2000 trees. A graph illustrates the Out-of-Bag (OOB) error rate of the Random Forest in relation to the number of trees. The Random Forest model is reconfigured using the ideal tree count, and feature significance is assessed via the “importance” function (45). Additionally, we cross-referenced the model genes with genes exhibiting differential expression identified using multi-omic analysis of BLCA from our previous publication, which analyzed urinary specimens from five BLCA cases compared to five healthy donors (46) and supplemented by additional proteomics from Zhang et al. (47). Ultimately we identified PSMB5 as the primary gene of interest.

We performed extensive analyses on PSMB5, encompassing gene expression profiling, assessment of survival probability, evaluation of progression-free survival, and clinical correlation studies. Additionally, we performed an extensive analysis of the immune landscape and tumor mutational burden to clarify immune-related characteristics and investigate possible implications for immunotherapy.

### MR and single-cell data analysis for PSMB5

To investigate the causal link between PSMB5 and BLCA, we used MR using Wald ratio methods (48). Exposure data comprised three single nucleotide polymorphisms (SNPs) from the eqtl-a-ENSG00000100804, filtered using a clumping window size of 10,000 Kb, R2<0.001, and F>10 (**Supplementary Table 1**). The outcome data was obtained from FinnGen (https://www.finngen.fi/en).

We employed single-cell RNA sequencing data from the GEO dataset GSE222315, which includes 9 BLCA cases and 4 surrounding normal tissue samples. Raw scRNA-seq data were converted into Seurat objects and underwent quality control according to specified thresholds to preserve high-quality cells: (1) Detection of 200–5,000 genes per cell; (2) Mitochondrial gene content not exceeding 15%; (3) Red blood cell gene expression rate surpassing 3%. Following normalization, batch effects were corrected using the Harmony integration method. Data was subjected to log-normalization and subsequently scaled using linear regression (49). Dimensionality reduction was performed using principal component analysis, followed by graph-based clustering via the “Find-clusters” algorithm (50). Visualization was conducted using UMAP, and the expression informed the annotation of various cell populations of classical marker genes (51). We examined the disparities in PSMB5 expression across different cell types and between the negative and positive groups. The correlation between PSMB5 expression and lymph node metastases was investigated in particular cell lines.

### Cell culture and transfection

All cell lines were acquired from the Gansu Province Clinical Research Center for Urinary System Diseases. SV-HUC-1 urethral epithelial cells were cultivated in Ham’s F12K medium, while BLCA cell (T24, UMUC-3, J82, 5637) were sustained in RPMI-1640 (Shanghai Yuanpei Biotechnology). Both media included 10% fetal bovine serum (FBS) from PAN Biotech and 1% penicillin-streptomycin at a concentration of 100 U/mL-100 μg/mL from Solarbio. Standard incubation conditions of 37 °C, 5% CO_2_, and humidity were maintained consistently.

The two small interfering RNAs (siRNAs) directed against PSMB5 were procured from Tsingke Biological, and the transfection reagent was sourced from Shanghai GenePharma Biotechnology (si1: 5’-CGAAAUGCUUCAUGGAACA-3’; si2: 5’-GGCAAUGUCGAAUCUAUGA-3’; si-NC: UUCUCCGAACGUGUCACGUTT). The efficacy of the knockdown was validated using western blot (WB) analysis at 48 hours post-transfection. Moreover, concurrent phenotypic experiments were conducted using the same procedure.

### Construction of overexpression cell line

The whole coding sequence of human PSMB5 was inserted into the pLV3-CMV-3×FLAG-mCherry-Puro vector (Miaoling Bio, China). HEK293T cells were co-transfected with psPAX2 and pMD2.G vectors, and the viral supernatant was harvested to infect J82 cells. Following puromycin selection, stable cell lines exhibiting PSMB5 overexpression were established. Cell transfection was performed using Polybrene (Solaibao, China) according to the manufacturer’s instructions.

### Western blotting

Total protein was extracted utilizing RIPA buffer (P0013B, Beyotime, China) augmented with protease inhibitors. Protein concentrations were measured via the Bicinchoninic Acid assay. After separation by SDS-PAGE electrophoresis, proteins were transferred to PVDF membranes. Membranes for immunoblotting were blocked with 6% non-fat dry milk and then treated with primary antibodies at 4 °C overnight. Protein bands were identified utilizing the Odyssey imaging system in conjunction with the appropriate secondary antibody (926-32211, Li-Cor, USA) for visualization. This work utilized the following antibodies: β-actin (cat#66009-1-Ig, Proteintech) and PSMB5 (cat#19178-1-AP, Proteintech).

### Cell counting kit-8

The Cell Counting Kit-8 (CCK8) was utilized to evaluate the proliferation. In accordance with the guidelines, cells (2 × 10³/well) were inoculated in 100 µL of media using 96-well plates, with three replicate plates established for various time points. CCK-8 reagent (AbMole BioScience) was applied at 10 µL per well at intervals of 0 to 96 hours. Following a 2-hour incubation, the optical density at 450 nm was assessed via a BioTek plate reader.

### Colony formation assay

For clonogenic tests, 6-well plates were inoculated with 1 × 10³ cells per well in 2 mL of medium. Following an 8–10 day cultivation at 37°C with 5% CO_2_, colonies were fixed with 4% PFA (Biosharp #BL539A), stained with 0.1% crystal violet (Solarbio #G1063), and subsequently photographed and quantified.

### Wound-healing assay

Transfected cells (6×10^5^) attained confluence 48 hours after transfection. Monolayers were scraped with sterile 200 μL tips, rinsed with PBS, and subsequently treated with serum-free media. Migration was evaluated by photographing wounds at 0 and 24 hours using inverted microscopy, with closure rates measured using ImageJ.

### Transwell migration assay

BLCA cells (1×10^5^ in 200 μL of serum-free media) were inoculated into LABSELECT chambers (8 μm holes; #14342). The lower chambers had 600 μL of RPMI-1640 enriched with 20% FBS as a chemoattractant. After 24–48 hours of incubation at 37 °C with 5% CO_2_, the transmigrated cells were subjected to methanol fixation (4%), crystal violet staining (0.1%; Solarbio #G1063), and subsequent microscopic counting.

### Cell apoptosis

Apoptosis was evaluated utilizing the Annexin V-FITC/PI kit (Multi Sciences #AP101) in accordance with the manufacturer’s specifications. Flow cytometric analysis (Beckman CytoFLEX S) was used to assess overall apoptosis by aggregating early and late apoptotic populations.

### Prediction of TCMs and molecular docking

To investigate the therapeutic potential of PSMB5 as a target, we employed the Coremine medical ontology information retrieval tool (www.coremine.com/medical/) to delineate PSMB5. Additionally, to obtain the target protein result files, the structures of TCMs were retrieved from PubChem (https://pubchem.ncbi.nlm.nih.gov/), while the structure of PSMB5 (PDB ID: 5l5w) was acquired from the PDB database (https://www.rcsb.org/). The requisite alterations to the receptor proteins, encompassing hydrogenation and charge equilibrium, were executed utilizing AutoDockTools 1.5.7 software. AutoDock Vina 1.1.2 was subsequently employed to mimic molecular docking between the pharmaceuticals and PSMB5 (52). The molecular docking results were visualized using PyMOL 3.1.5.1, focusing on high-affinity complexes.

### Statistical analysis

Statistical analyses were conducted using R (v4.3.3) and GraphPad Prism (v9.0). Continuous variables were compared between groups using either Student’s t-test (parametric) or Wilcoxon rank-sum test (non-parametric), based on normality assessment. Categorical variables were evaluated with the χ² test or Fisher’s exact test, chosen based on anticipated cell frequencies. Survival outcomes were evaluated with Kaplan-Meier estimation and log-rank testing for group comparisons, augmented by multivariate Cox proportional hazards regression. All experimental techniques were conducted in three biological replicates, with data presented as mean ± standard deviation (SD). Statistical significance was determined at *p* < 0.05, with asterisk notation indicating non-significant (n.s.); *p ≤ 0.05; **p ≤ 0.01; ***p ≤ 0.001; ****p ≤ 0.0001. *P values* below 0.05 were considered statistically significant (53).

## Result

### Identification and functional characterization of differentially expressed genes linked to PANoptosis in BLCA

**Figure 1:**
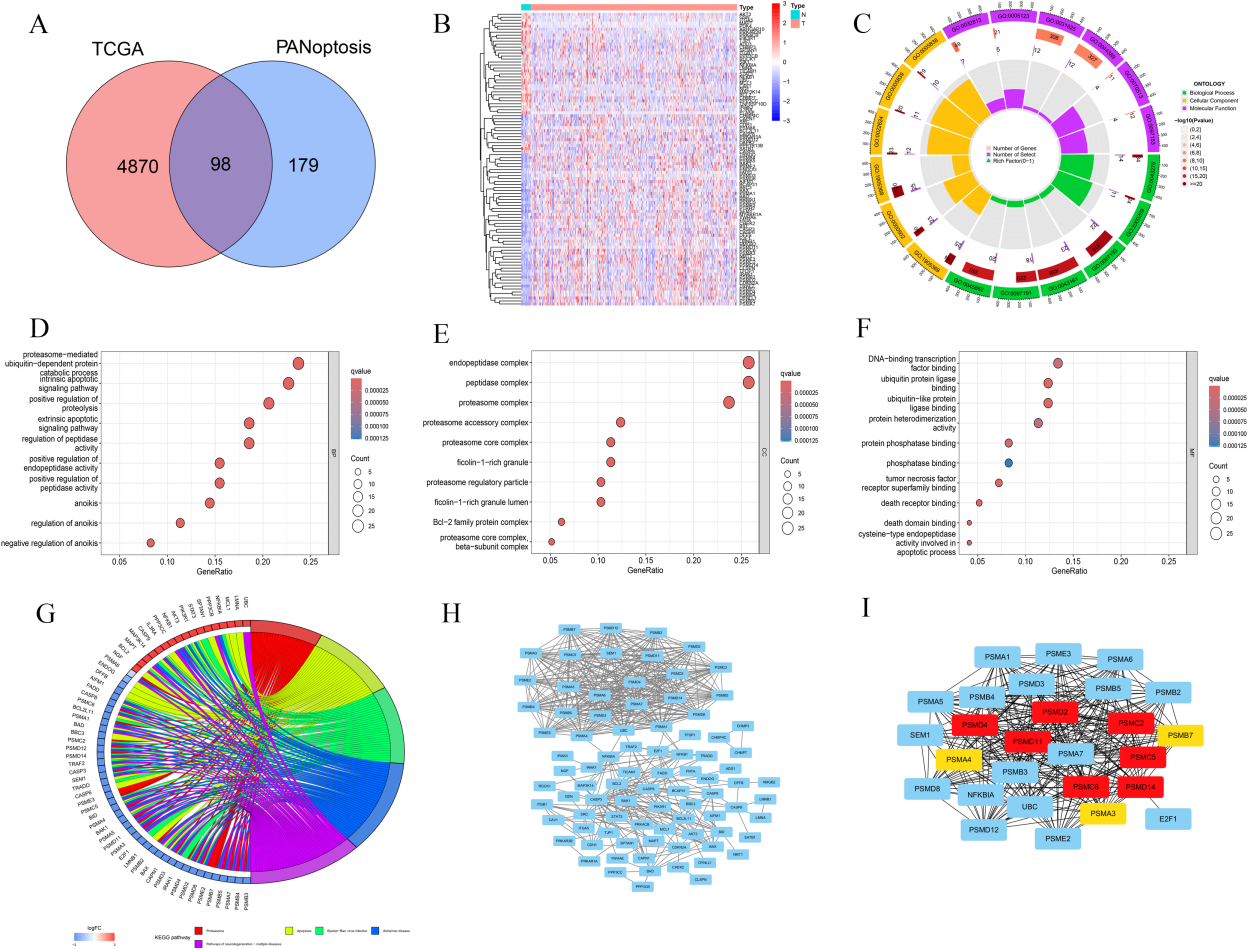
Identification and functional analyses for the BLCA-PANs. **(A)** Venn diagram shows 98 BLCA-PANs overlapping PANoptosis and differential BLCA genes. **(B)** Heatmap shows 98 BLCA-PANs between BLCA and normal patients. **(C)** Circle chart shows GO enrichment analysis. **(D-F)** Bubble charts indicates the main enrichment functions. **(G)** KEGG analysis shows 5 pathway enriched by the BLCA-PANs. **(H, I)** PPI and 10 core genes.

We found 4,968 differentially expressed genes in BLCA. These were compared with 277 PANoptosis-related genes sourced from the literature, resulting in the identification of 98 BLCA-PANs (**Figures 1A, B**). Gene Ontology (GO) analysis revealed the abundance and enrichment significance of BLCA-PANs across various levels (**Figure 1C**). The biological process (BP) exhibited significant enrichment in proteasome-mediated ubiquitin-dependent protein degradation (**Figure 1D**). The cellular component (CC) revealed that the principal enrichment functions of BLCA-PANs were endopeptidase, peptidase, and proteasome complexes (**Figure 1E**). The molecular function (MF) exhibited significant enrichment in DNA-binding transcription factor interactions and ubiquitin-related ligase interactions (**Figure 1F**). The Kyoto Encyclopedia of Genes and Genomes (KEGG) indicated that BLCA-PANs were predominantly abundant in the proteasome and apoptotic pathways (**Figure 1G**). Furthermore, we conducted a PPI analysis to demonstrate the interactions of BLCA-PANs and identified several key genes primarily associated with the proteasome subunit family (**Figures 1H, I**).

### Prognosis, immunological profiles, and mutational landscapes in BLCA-PANs distinct subtypes

**Figure 2:**
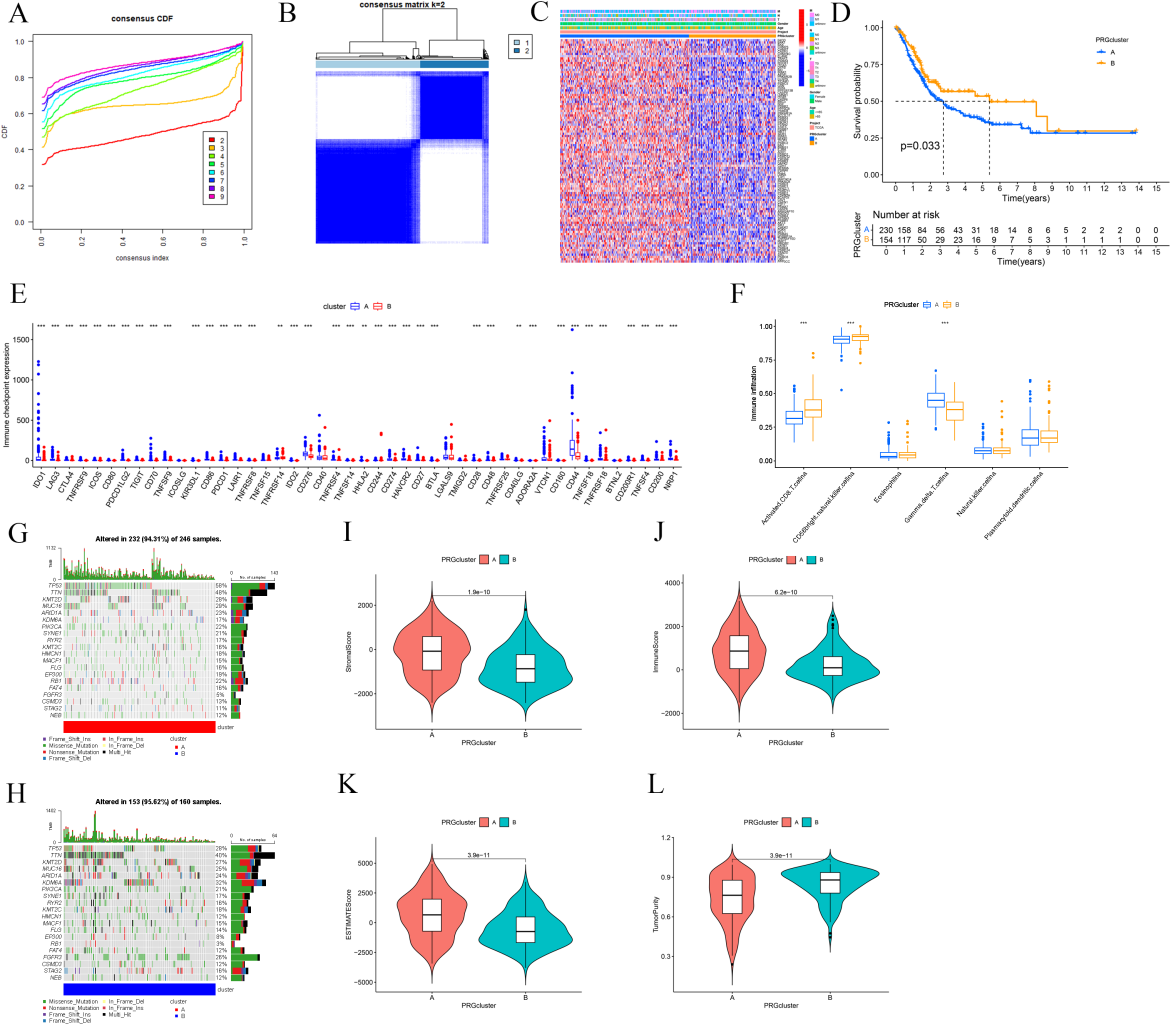
Clinical and immunological difference between the two molecular subtypes. **(A)** CDF curves assess average consistency. **(B)** Patients were divided into two molecular subtypes. **(C)** Heatmap shows clinical characterizations. **(D)** Survival analysis between the two subtypes. **(E)** Differential expression of ICs. **(F)** Immune infiltration analysis with ssGSEA. **(G, H)** Waterfall charts of TMB shows mutated genes for the two subtypes. **(I-L)** TME score includes Stromal Score, immune Score, ESTIMATE Score, and Tumor Purity. ** p ≤ 0.01; *** p ≤ 0.001.

Consensus clustering analysis was performed on BLCA-PANs expression patterns to categorize patients into two distinct subtypes: Cluster A (n = 246) and Cluster B (n = 160) (**Figures 2A, B**). A heatmap was later generated to depict the differential expression of BLCA-PANs concerning molecular subtypes (Cluster A/B) and clinicopathological characteristics, including gender, age, and staging factors (T, N, M) (**Figure 2C**). OS analysis indicated that Cluster A demonstrated a markedly inferior overall survival probability relative to Cluster B (*p* = 0.033; **Figure 2D**). The study revealed that most ICs were considerably overexpressed in Cluster A, while only a select few showed elevated expression in Cluster B (*p* < 0.05; **Figure 2E**). Immune infiltration with ssGSEA indicated that Cluster A exhibited a statistically significant prevalence of γδ T cells, while Cluster B had a predominant infiltration of activated CD8 T cells and CD56bright natural killer cells (*p* < 0.05; **Figure 2F**). Waterfall charts of TMB indicated that Cluster A displayed elevated gene mutation rates compared to Cluster B (**Figures 2G, H**). Furthermore, the TMB score values indicated a significant difference between the two clusters (*p* < 0.05; **Figures 2I-L**).

### Creation and internal validation of a prognostic risk score model based on BLCA-PANs

**Figure 3:**
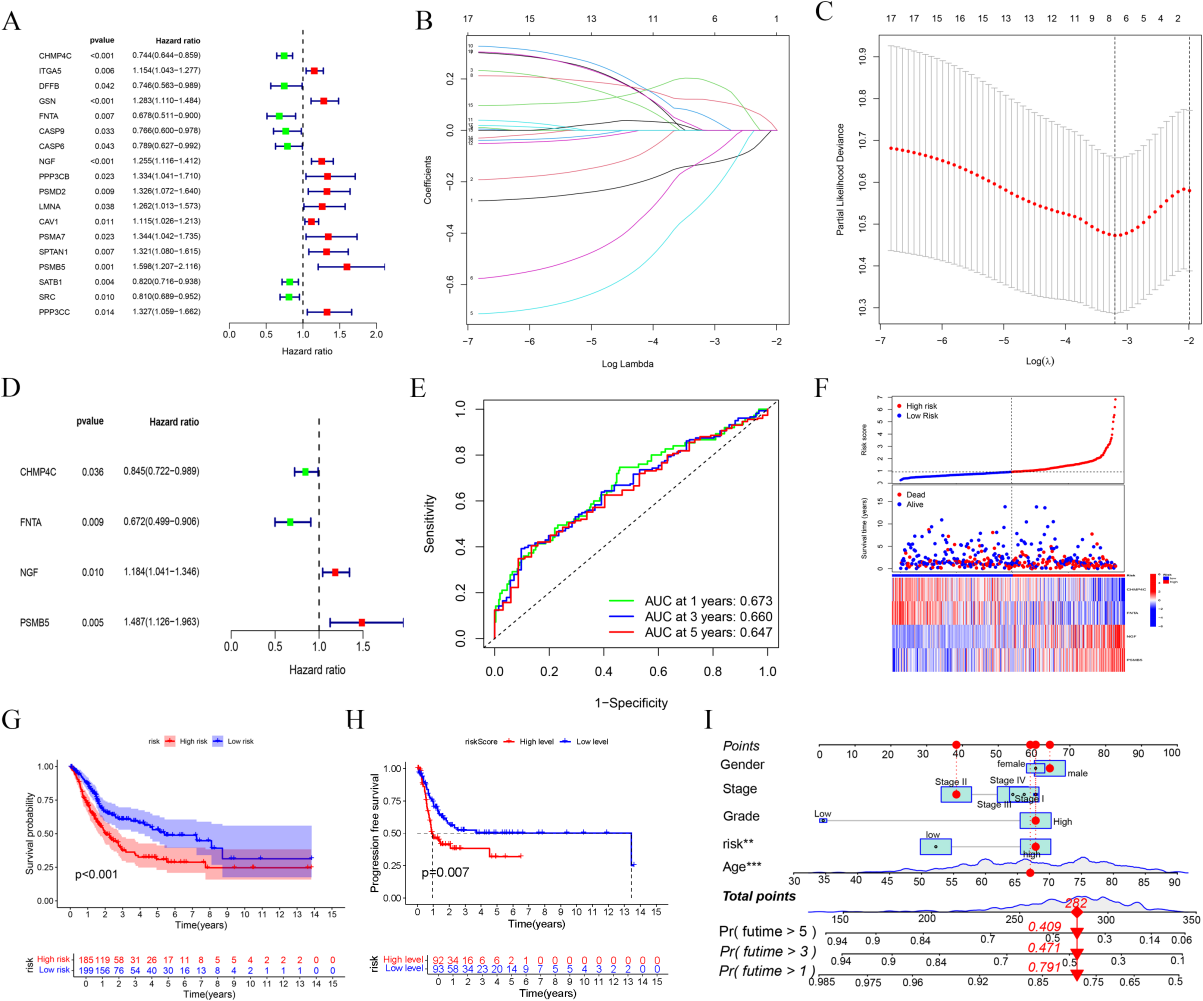
Prognostic model based on BLCA-PANs. **(A)** Forest map shows the result of univariate Cox regression. **(B, C)** The process of LASSO regression. **(D)** Forest plot presents four genes selected for risk scoring model. **(E)** ROC curves at 1, 3, 5 years. **(F)** Distribution of all patients. **(G, H)** OS and PFS analyses between high-risk and low-risk groups. **(i)** Nomogram with clinical characterizations.

Employing Cox regression studies, we developed a prediction model comprising four BLCA-PANs by LASSO regression (**Figures 3A-D**). The model exhibited enhanced predictive accuracy relative to the clinical baseline, as evidenced by C-index, AUC curve, and DCA analyses (**Supplementary Figures 1A-C**). The Receiver Operating Characteristic (ROC) curves demonstrated the model’s prognostic capability (**Figure 3E**). Based on the computed risk scores in BLCA (BLCA-Riskscore), patients were classified into High- and Low-risk categories (**Figure 3F**). Marked enhancements in OS (*p* < 0.001) and PFS (*p* = 0.007) were noted in the Low-risk group (**Figures 3G, H**). As a result, we constructed a nomogram (**Figure 3I**) and a standard curve (**Supplementary Figure 1D**).

**Figure 4:**
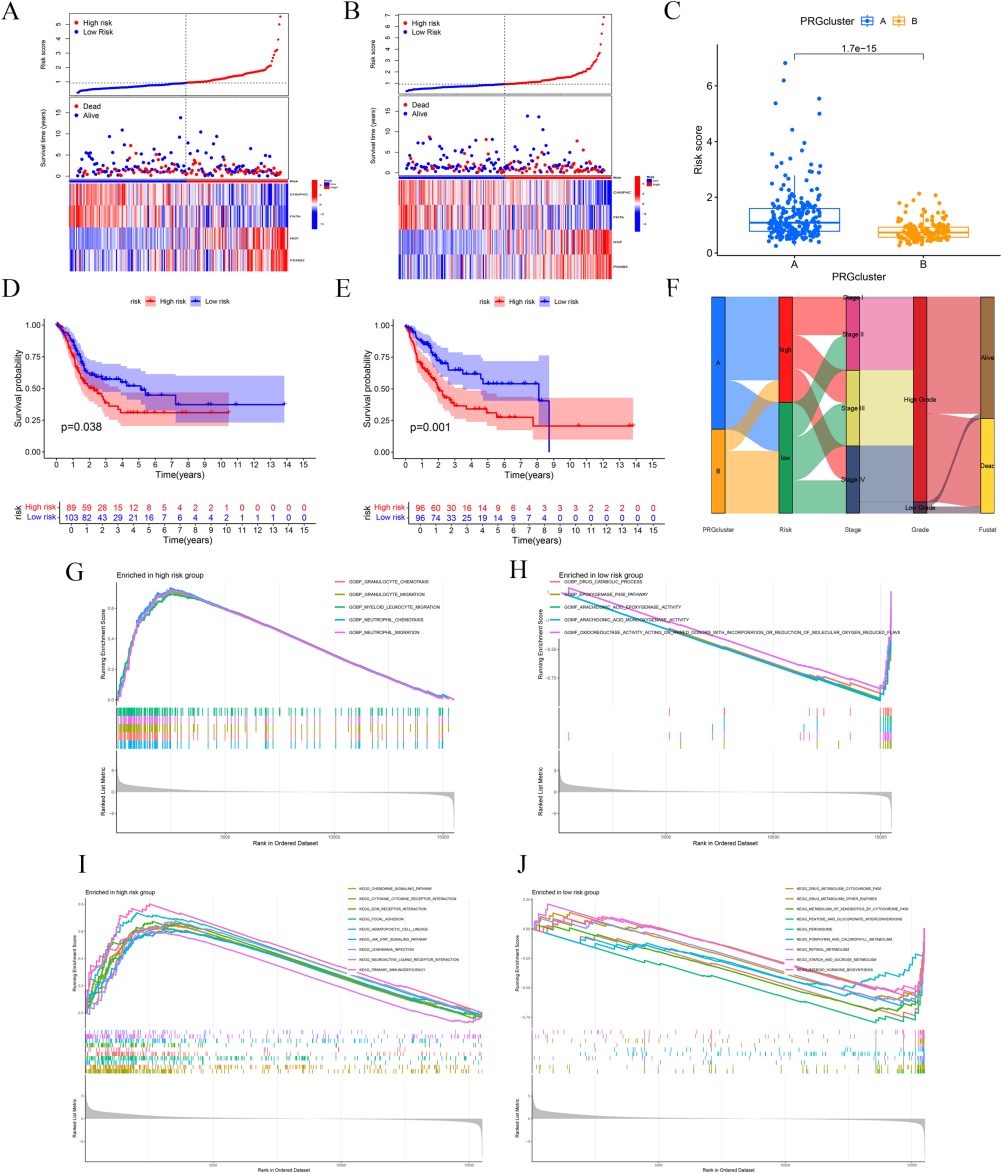
Internal validation and functional enrichment with the model. **(A, B)** Test and train sets. **(C)** A significant difference between Cluster A and B **(D, E)** OS analysis of the two sets. **(F)** Sankey diagram shows associations between the model and clinical data. **(G-J)** GSEA analyses with high-risk and low-risk groups.

To validate the model’s credibility and consistency, we partitioned the TCGA database into training and testing sets (**Figures 4A, B**). The operating system results for the two sets were consistent with the prior findings (*p-value* for test set = 0.038, *p-value* for train set = 0.001; (**Figures 4D, E**). Utilizing the BLCA-Riskscore to evaluate Clusters A and B, Cluster A had markedly higher risk scores compared to Cluster B, correlating with inferior overall survival rates in this cohort (**Figure 4C**). The Sankey diagram was used to illustrate the relationship between the groups and clinical features (**Figure 4F**). Furthermore, we conducted GSEA analyses for high-risk and low-risk groups based on GO and KEGG. The high-risk group was primarily characterized by the chemotaxis and migration of granulocytes and neutrophils, as well as the interaction with extracellular matrix receptors and the activation of the JAK-STAT signaling pathway. The low-risk group was linked to the epoxygenase P450 pathway, arachidonic acid epoxygenase or monooxygenase activity, and so on(**Figures 4G-J**).

### Somatic mutation profiles and immune micro-environment features among BLCA-Riskscore categories

**Figure 5:**
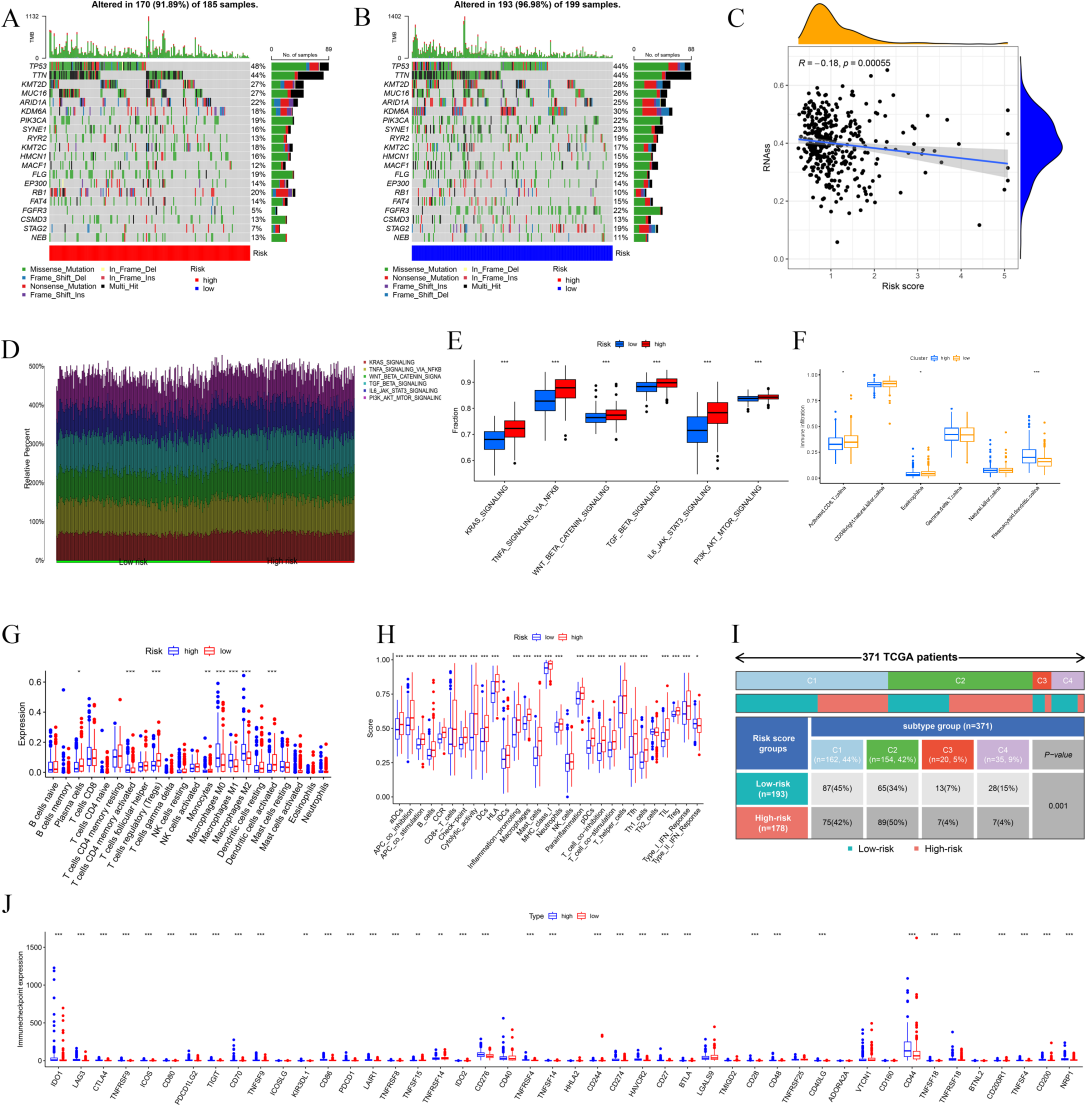
Immune landscape of High-risk and Low-risk groups. **(A, B)** Waterfall charts show mutated genes for the two groups. **(C)** The correlation between risk score and RNAss. **(D, E)** Differential immune-related signaling pathways between High-risk and Low-risk groups. **(F)** Immune infiltration based on the ssGSEA algorithm between high- and low-risk groups. **(G, H)** Immune cell expression and immune function analyses. **(I)** Immune subtypes analysis based on the TCGA. **(J)** Differential analyses of ICs. * p ≤ 0.05; ** p ≤ 0.01; *** p ≤ 0.001.

The cascade charts illustrated the disparity in mutational landscapes between high-risk and low-risk groups (**Figures 5A, B**). The association investigation indicated a small inverse correlation between RNAss and risk score, implying diminished stemness characteristics (**Figure 5C**). We identified statistically significant dysregulations in pathways, notably impacting the KRAS cascade, NF-κB-mediated TNF-α signaling, β-catenin-dependent WNT pathway, TGF-β transduction, IL-6-JAK-STAT3 axis, and the PI3K-AKT-mTOR network (**Figures 5D, E**).

According to ssGSEA, plasmacytoid dendritic cells (pDC) exhibited substantial immunological infiltration in the high-risk cohort. Moreover, CD8^+^ T cells and CD56^brilliant^ natural killer (NK) cells were significantly infiltrated in the low-risk cohort (**Figure 5F**). The high-risk cohort had significant expression of M0, M1, and M2 macrophages, corresponding with specific immunological activity patterns. Conversely, the low-risk group exhibited a predominance of immunosuppressive regulatory T cells (Tregs), antibody-secreting plasma cells, monocytic lineage cells, and activated dendritic cell populations (**Figure 5G**). The examination of immune function indicated that immune responses were predominantly heightened in the high-risk group, encompassing APC co-inhibition, APC co-stimulation, MHC-I, neutrophils, para-inflammation, T cell co-inhibition, T cell co-stimulation, Th1 cells, Th2 cells, tumor-infiltrating lymphocytes (TILs), Tregs, and Type-I interferon response. Only the Type-II IFN response is considerably elevated in the low-risk group (**Figure 5H**). A cohort of 371 BLCA patients was categorized into four clusters (C1, C2, C3, C4) and classified as high-risk or low-risk based on established BLCA-Riskscore thresholds. A statistically significant difference was noted between risk strata using the chi-square test (**Figure 5I**). The comparative study of IC expression profiles differentiated the high-risk group from the low-risk group. Most ICs exhibited considerable differential expression among cohorts (**Figure 5J**).

**Figure 6:**
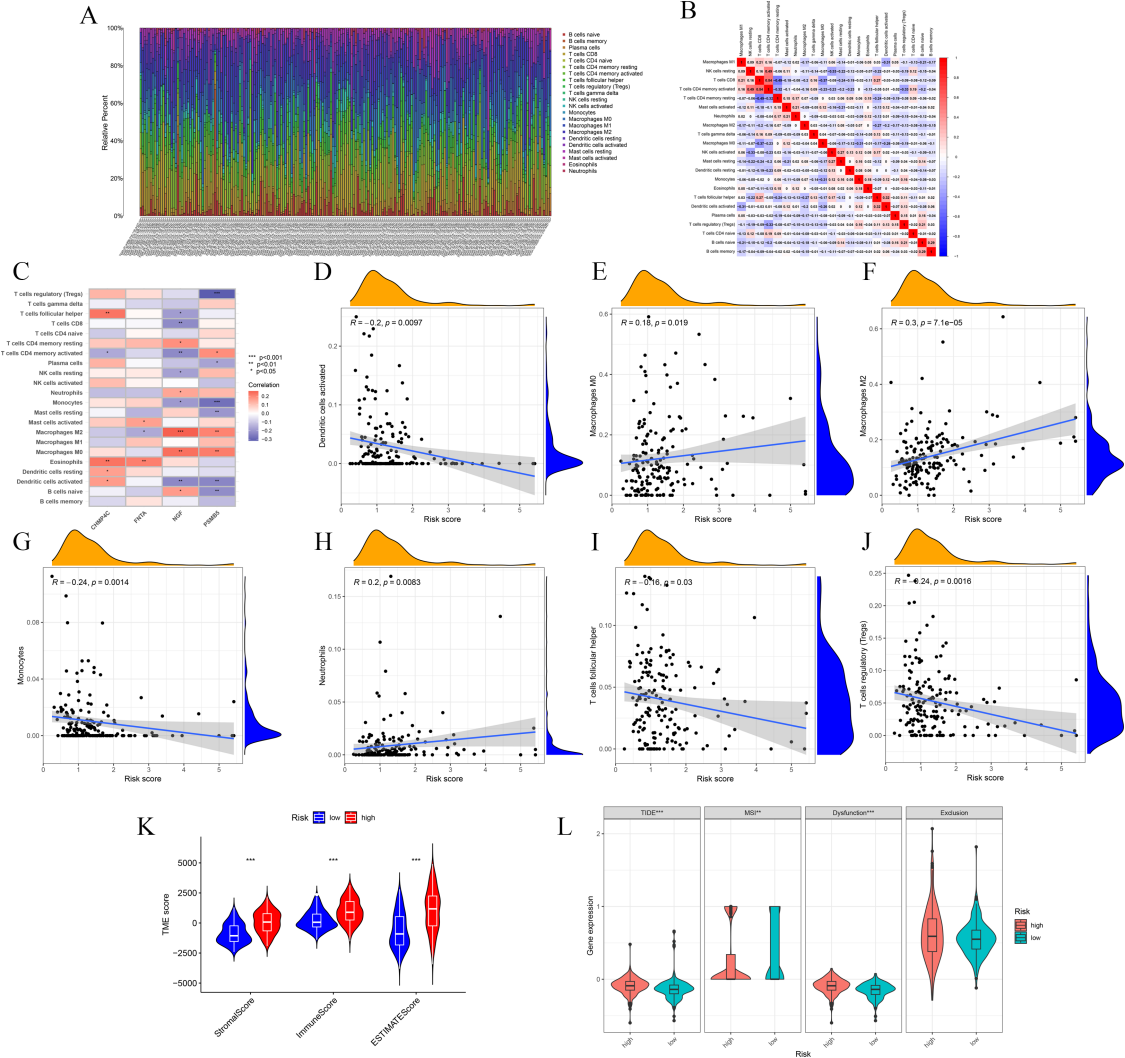
Correlation analyses of immune cells and TME with the risk groups. **(A-C)** Immune cells exhibit meaningful connections with risk score and model genes. **(D-J)** Plots show significant correlations between seven immune cells and risk score. **(K)** Box plot shows TME scores between High-risk and Low-risk groups. **(L)** TIDE, MSI, Dysfunction and Exclusion analyses. * p ≤ 0.05; ** p ≤ 0.01; *** p ≤ 0.001.

Analysis of immune cell correlations indicated that seven immune cell types were significantly associated with the BLCA-Riskscore, comprising three positively correlated (M0, M2, Neutrophils) and four negatively correlated [Dendritic cells (activated), Monocytes, T follicular helper cells (Tfh), Tregs] cell types (**Figures 6A-J**). The TME score exhibited notable disparities (**Figure 6K**). Furthermore, assessments of TIDE and MSI indicated that the TIDE score, MSI status, and Dysfunction score, excluding the Exclusion score, exhibited considerable variance specific to the cohort (**Figure 6L**). Ultimately, drug susceptibility analysis revealed that 29 medications exhibited a significant correlation (**Supplementary Figure 2**).

### Machine learning identifies the key feature gene

**Figure 7:**
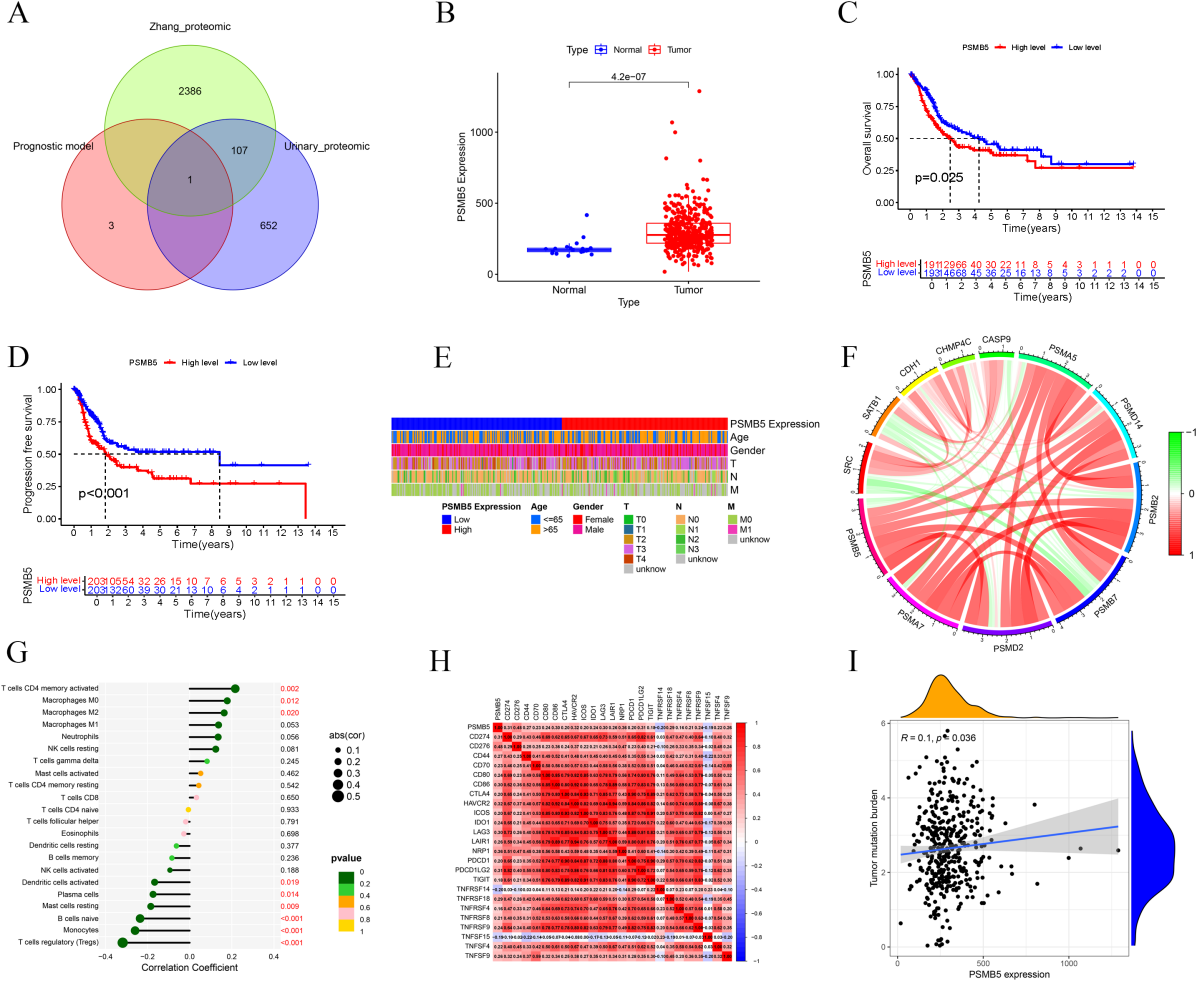
Clinical and immunological analyses of PSMB5. **(A)** Venn diagram shows PSMB5 overlapping the model and two datasets. **(B)** Expression of PSMB5 between normal and tumor patients. **(C, D)** OS and PFS analyses of PSMB5. **(E)** Heatmap shows associations between PSMB5 and clinical parameters. **(F)** Interaction of PSMB5 with other genes. **(G)** Immune cells correlation analysis of PSMB5. **(H)** Association analysis of PSMB5 with ICs. **(I)** Positive relationship between PSMB5 expression and TMB.

To advance research on BLCA-PANs, we employed four machine learning techniques and integrated the results of Zhang et al. with our urine proteomics data to identify critical feature genes (**Figure 7A**). The Boruta algorithm demonstrates that PSMB5 attained the highest score (**Supplementary Figures 3A, B**). SVM-RFE indicates that PSMB5 is the nearest to the scatter point, exhibiting the highest average ranking (**Supplementary Figure 3C**). PSMB5 demonstrates the most excellent absolute coefficient value in the Lasso regression model (**Supplementary Figure 3D**). Random Forest demonstrates the most excellent Mean Decrease Gini score (**Supplementary Figures 3E, F**). The essential gene PSMB5 was ultimately acquired.

### Clinical and immunological correlation, MR, and single-cell analysis of PSMB5

Differential expression analysis revealed that PSMB5 was markedly overexpressed in BLCA patients (**Figure 7B**). The studies of OS (*p* = 0.025) and PFS (*p* < 0.001) demonstrated that PSMB5 was significantly associated with clinical prognosis (**Figures 7C, D**). A heatmap illustrating the relationship between PSMB5 and clinical characteristics was generated (**Figure 7E**). Subsequently, we examined the connection between PSMB5 and other BLCA genes (**Figure 7F**). Analysis of immune cells yielded results consistent with the high-risk and low-risk groups, correlating with M0, M2, Monocytes, and Tregs (**Figure 7G**). Furthermore, we identified several ICs that were statistically significant with PSMB5 (**Figure 7H**). The TMB exhibited a positive correlation with PSMB5 expression (*p* = 0.036; **Figure 7I**).

Furthermore, we identified three SNPs in PSMB5 (rs12590429, rs117058979, rs11543947) to conduct MR. Results identified rs117058979 as a causative variant for BLCA [OR = 2.267 (1.008, 5.097), *p* = 0.048] (**Supplementary Table 2**).

**Figure 8:**
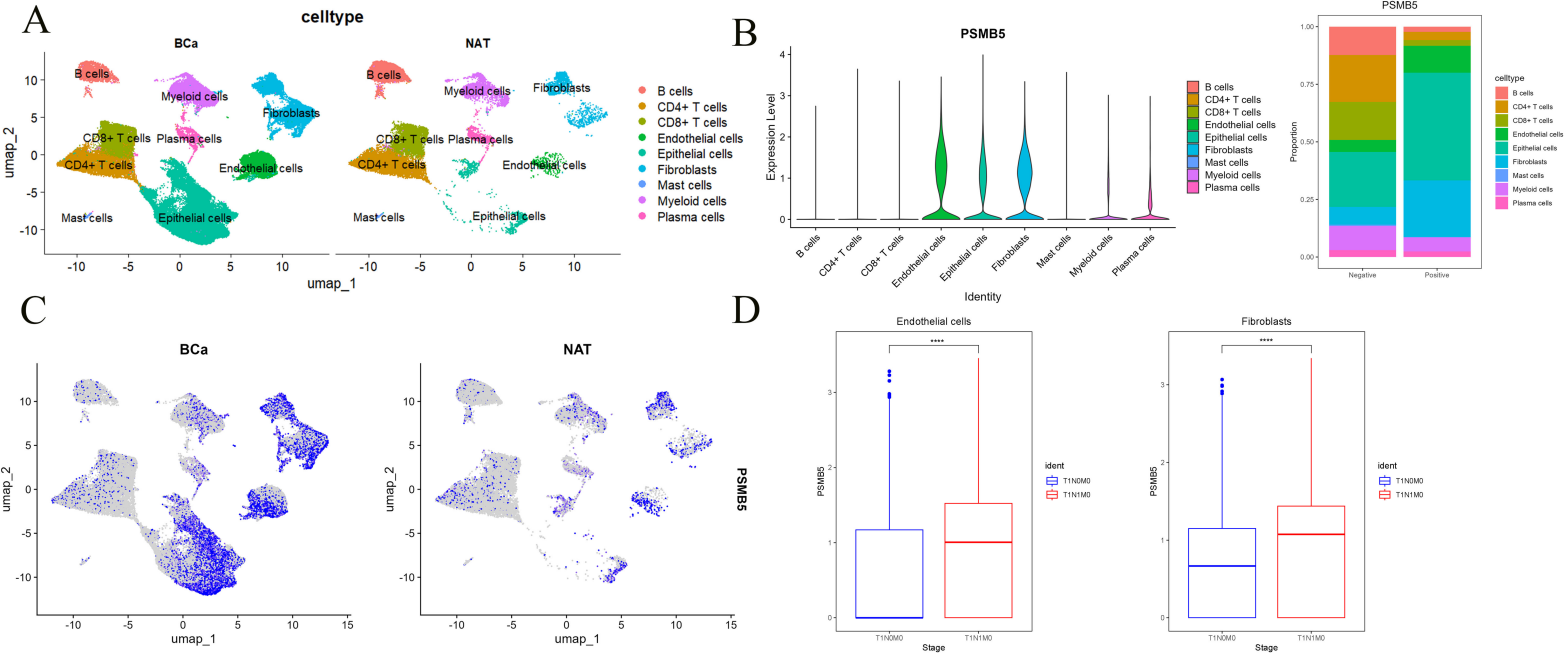
Single-cell analysis of PSMB5. **(A)** Clustering of cells in BLCA and normal groups. **(B)** Proportion of PSMB5 in 9 cell lines. **(C)** Expression of PSMB5 in BLCA and normal groups. **(D)** Differential analyses between PSMB5 expression and lymph node metastasis in Endothelial cells and Fibroblasts. **** p ≤ 0.0001.

Upon normalizing and annotating the single-cell database, the BLCA group and the normal group predominantly clustered into nine categories of cell lines (**Figure 8A**). PSMB5 exhibited markedly elevated expression in the BLCA cohort, predominantly among Endothelial cells, Epithelial cells, and Fibroblasts (**Figures 8B, C**). Consequently, we meticulously analyzed the relationship between PSMB5 expression and lymph node metastasis in all three cell lines, discovering substantial statistical differences for endothelial cells (*p* < 0.0001) and fibroblasts *(p* < 0.0001) (**Figure 8D**).

### The impact of knockdown and overexpression of PSMB5 on the biological behavior of BLCA cells

**Figure 9:**
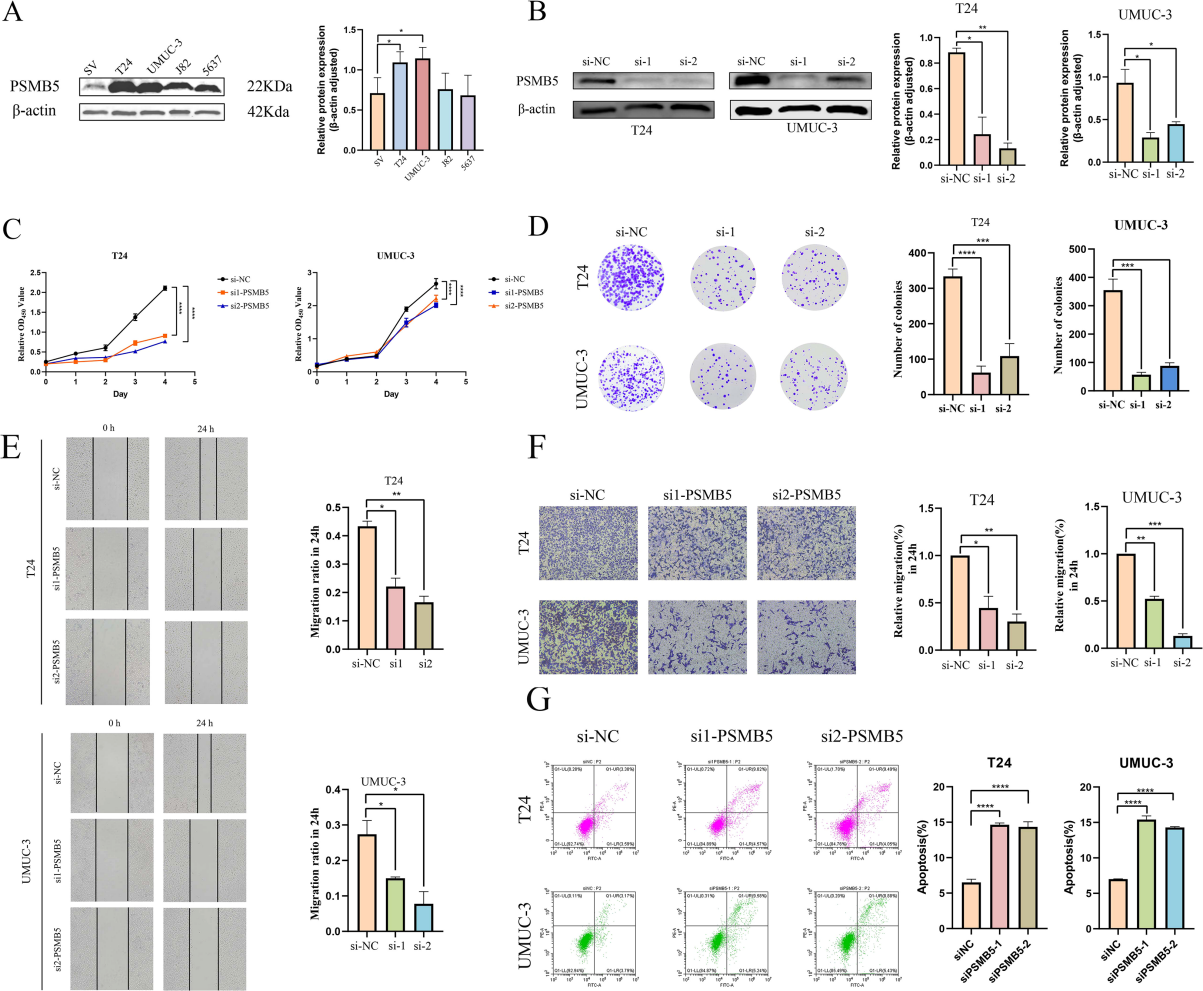
*In vitro* experiments with PSMB5 knockdown. **(A)**. Expression levels of PSMB5 in SV, T24, UMUC-3, J82 and 5637 cell lines. **(B)** Knockdown of PSMB5 in T24 and UMUC-3. **(C)** CCK-8 proliferation assay. **(D)** Colony formation experiment. **(E)** Wound-healing assay. **(F)** Trans-well migration assay. **(G)** Flow cytometric analysis of apoptosis. All experimental techniques were conducted in three biological replicates with asterisk notation indicating non-significant (n.s.); *p ≤ 0.05; **p ≤ 0.01; ***p ≤ 0.001; ****p ≤ 0.0001.

WB analysis revealed distinct expression profiles of PSMB5 across various BLCA cell lines, with significantly increased expression levels observed in T24 and UMUC-3 cells (**Figure 9A**). In these two cell lines, siRNA transfection resulted in a knockdown efficiency of about 60% for PSMB5. Subsequently, comprehensive *in vitro* functional studies were performed. (**Figure 9B**). The CCK-8 proliferation assay and analysis of colony formation consistently indicated that PSMB5 depletion markedly reduced cellular proliferation compared to the negative control (NC) groups (**Figures 9C, D**). Additionally, both wound-healing and transwell migration experiments demonstrated significantly reduced migratory ability in PSMB5-knockdown cells compared to controls (**Figures 9E, F**). Flow cytometric examination of apoptosis revealed that silencing PSMB5 markedly increased apoptotic rates compared to the NC groups (**Figure 8G**). After overexpressing PSMB5 in J82 with an overexpression efficiency of about 40%, the opposite biological behavior was displayed (**Supplementary Figure 4A**). The abilities of proliferation and migration are enhanced, and cell apoptosis is significantly reduced (**Supplementary Figures 4B-F**).

### TCMs prediction analysis

**Figure 10:**
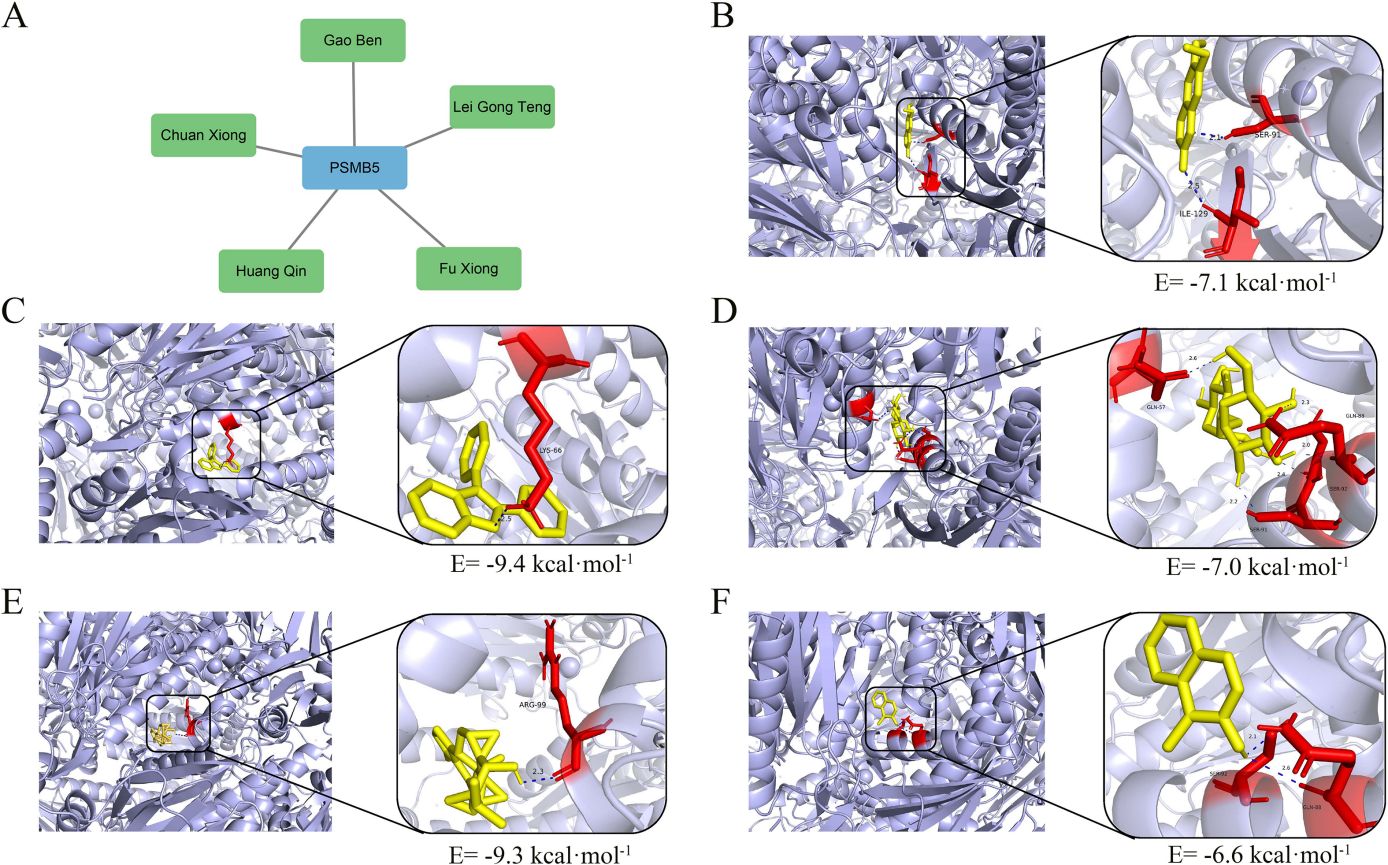
TCMs prediction and molecular dockings. **(A)** Five TCMs targeting PSMB5. **(B)** Molecular docking of Chuanxiong, binding energy= -7.1 kcal·mol^-1^. **(C)** Molecular docking of Gao Ben, binding energy= -9.4 kcal·mol^-1^. **(D)** Molecular docking of Fu Xiong, binding energy= -7.0 kcal·mol-1. **(E)** Molecular docking of Lei Gong Teng, binding energy= -9.3 kcal·mol^-1^. **(F)** Molecular docking of Huang Qin, binding energy= -6.6 kcal·mol^-1^.

We identified five TCMs related to PSMB5 from the Coremine dataset: Chuanxiong Rhizoma (Chuan Xiong in Chinese), Ligusticum sinense Oliv. (Gao Ben in Chinese), Fuxiong Rhizome (Fu Xiong in Chinese), Tripterygium wilfordii Hook. f. (Lei Gong Teng in Chinese), and Scutellaria baicalensis Georgi (Huang Qin in Chinese) (**Figure 10A**). Subsequently, molecular dockings were performed, revealing that binding energies below -5.0 kcal·mol^-^¹ indicated increased molecular affinity (**Figures 10B-F**). These interactions offer a potential pathway for further investigation into the use of TCMs in the treatment of BLCA.

## Discussion

Recent investigations have identified PANoptosis as strongly associated with diverse oncogenesis in several malignancies, including gastric cancer, colorectal cancer, and prostate cancer (54–57). In the study, we methodically performed consensus clustering on filtered BLCA-PANs and created the BLCA-Riskscore to formulate a predictive model. The cohort was divided into two separate clusters matching the BLCA-Risk score groups. Subsequent analyses assessed prognostic disparities, characteristics of the immunological microenvironment, and mutational landscapes among these clusters and risk categories. Analysis of differential expression of immune checkpoints suggested potential targets for IC inhibitors in high-risk groups. At the same time, drug sensitivity profiling indicated increased therapeutic responses to several drugs in high-risk patients. The large intergroup differences observed strongly substantiated the PANoptosis-based classification technique. This classification presents a molecular framework for studying PANoptosis-related processes in BLCA and suggests possible treatment targets. Significantly, the findings demonstrated that PANoptosis regulates BLCA heterogeneity, providing therapeutically relevant insights for enhancing personalized therapy strategies (58).

GSEA analysis indicated that PANoptosis-related genes affect BLCA progression, stem cell preservation, invasion, and therapeutic resistance via modulating pathways including Wnt/β-catenin, TNF-α/NF-κB, KRAS and so on. Research has demonstrated that the deletion or mutation of the PTEN gene is a common occurrence in BLCA (59). Inactivation of PTEN results in substantial buildup of PIP3, thus activating the PI3K/AKT/mTOR pathway. This route modulates BLCA proliferation by suppressing pro-apoptotic proteins (e.g., Caspase-9), enhancing glycolysis in neoplastic cells, and boosting angiogenesis (60). In advanced phases, TGF-β facilitates tumor invasion and metastasis by triggering the epithelial-mesenchymal transition (EMT), fostering an immunosuppressive environment, and promoting angiogenesis. The activation of the IL-6/JAK/STAT3 pathway enhances the expression of cell cycle-promoting proteins, including Cyclin D1 and c-Myc, as well as anti-apoptotic proteins such as Bcl-2 and Bcl-xL (61). This facilitates the proliferation of BLCA cells and contributes to their resistance to apoptosis induced by therapies such as chemotherapy and radiotherapy. Concurrently, pathway activation stimulates the expression of vascular endothelial growth factor (VEGF) and enhances stromal markers, including N-cadherin and vimentin, thereby facilitating tumor growth and metastasis (62). Additionally, it inhibits the activity of CD8 T cells and helper T cells, attracts myeloid-derived suppressor cells (MDSCs) and Tregs, and enhances PD-L1 expression on both tumor and immune cells. These acts jointly promote the establishment of an immunosuppressive microenvironment (63).

Our examination of immune infiltration revealed that the levels of CD8^+^ T cells and CD56^brilliant^ NK cells were markedly elevated in the favorable prognosis B cluster and low-risk groups, corroborating existing research on immune cells. CD8^+^ T cells directly eliminate tumor cells by identifying tumor antigens, such as peptide fragments presented by MHC class I molecules (64). The granzyme and perforin they release can then trigger tumor cell apoptosis. Furthermore, these cells establish immunological memory inside the tumor microenvironment, sustaining prolonged anti-tumor responses and diminishing the likelihood of recurrence. Prior research indicates that BLCA patients exhibiting elevated CD8^+^ T cell infiltration demonstrate improved responses to PD-1 medications (65). The CD56^bright^ NK cell fraction predominantly secretes cytokines (IFN-γ and TNF-α), which augment antigen presentation by stimulating macrophages and dendritic cells, thereby facilitating T cell infiltration (66). They exhibit elevated expression of CD16 and NKG2D receptors, which are capable of identifying stress ligands on tumor cells (such as MICA/B) and function synergistically with CD8^+^ T cells to eradicate immuno-evasive tumor cells. Research has established that CD56^bright^ NK cells signify a favorable prognosis for patients with BLCA. Conversely, γδ T cells may facilitate tumor angiogenesis and stroma remodeling by secreting cytokines such as IL-17 and IL-22. pDCs within the tumor microenvironment inhibit the anti-tumor functions of CD8^+^ T cells and NK cells by releasing immunosuppressive cytokines (IL-10 and TGF-β) and promoting the proliferation of Tregs (67). Moreover, pDCs can directly suppress effector T cell activities and facilitate tumor immune evasion by expressing immune checkpoint molecules, such as PD-L1 (68).

By using various machine learning methods to screen for important feature genes (69), combined with previous proteomics data, we found that PSMB5 is a significantly upregulated oncogenic gene. MR further validated that PSMB5 is a crucial pro-cancer factor for BLCA. Its functional relevance in BLCA remains notably unexamined. Therefore, it is imperative to investigate the processes in BLCA related to PSMB5. Single-cell profiling revealed enrichment of PSMB5 in endothelial cells, epithelial cells, and fibroblasts, while PSMB5 overexpression exhibited a substantial correlation with lymph node metastases. Our validation experiments verified the overexpression of PSMB5 in BLCA. Subsequent *in vitro* functional tests demonstrated that PSMB5 knockdown significantly impeded tumor cell growth and migration while markedly promoting apoptosis. Five TCMs targeting PSMB5 demonstrated considerable therapeutic efficacy.

PSMB5 is one of the 17 critical subunits of the 20S core particle β-subunit family (70). The beta type-5 subunit of the proteasome co-assembles with other β-subunits to create two heptameric rings that comprise the proteolytic compartment responsible for substrate cleavage (71, 72). This subunit is crucial for the development of the 20S proteasome and is functionally involved in ubiquitin-dependent proteolysis (73). This pathway is accountable for the deterioration of approximately 80% of proteins within cells in eukaryotes and demonstrates a substantial association with apoptosis (74). PSMB5 is mechanistically associated with oncogenesis in several malignancies, especially in breast, prostate, and esophageal cancers (75). The correlation between the expression levels of this subunit and tumor cell resistance to chemotherapeutic agents is particularly significant (76, 77). The findings suggest that further research on PSMB5 may reveal new pathways involved in bladder carcinogenesis, and that targeted suppression of PSMB5 expression could potentially enhance tumor cell sensitivity to chemotherapy. The exact regulatory mechanisms linking the ubiquitin-proteasome system to apoptosis, as well as the molecular pathways by which PSMB5 affects chemosensitivity, are not fully understood and require further investigation.

Moreover, the most prominent characteristic of TCM that can efficiently activate or suppress PANoptosis is the synergistic process involving several components and targets (78). The distinctive mechanism of “network pharmacology” allows traditional Chinese medicine to demonstrate considerable benefits in intricate pathological states, including tumors and inflammatory disorders. The aqueous extract of Achyranthes aspera mitigates cisplatin-induced nephrotoxicity by regulating PANoptosis, thereby maintaining tubular integrity (79). Chlorogenic acid produced from Yinhua Pinggan Granules demonstrates dual antioxidative and anti-inflammatory properties, mitigating macrophage PANoptosis triggered by drug-resistant E. coli (80). This multi-target intervention is crucial, as it can prevent treatment resistance resulting from single-pathway restriction and offers a novel approach to addressing tumor heterogeneity and microenvironment adaptation. The coadministration of cisplatin and berberine synergistically enhances the lethality of ovarian cancer cells by simultaneously activating apoptosis and necroptosis, thereby enhancing chemotherapeutic efficacy (81). We identified five TCMs, namely Chuan Xiong, Gao Ben, Fuxiong, Lei Gong Teng, and Huang Qin, which have therapeutic potential for BLCA, improve patient prognosis and indicate prospective avenues for further research.

We recognize multiple limitations in our present investigation. We recognize some limitations in the present investigation. This study relies exclusively on the TCGA database, where the disparity between normal and tumor samples may compromise the efficacy of detecting differentially expressed genes, and there is a lack of external cohort validation. Future research should augment the quantity of normal samples, enhance unbalanced learning algorithms, and do external validation. Furthermore, it is essential to validate the biological functions of the 98 differentially expressed genes using tumor samples or animal models. The predictive model employs only LASSO regression, which may result in lower AUC values. Future investigations may integrate supplementary machine learning algorithms (82). Only one SNP demonstrated a probable causal link with BLCA, hence precluding sensitivity and heterogeneity studies. The molecular mechanisms underlying PANoptosis between PSMB5 and BLCA progression remain to be fully elucidated through experimental validation. Moreover, although the expected TCMs were validated by molecular dockings, their fundamental associations on PSMB5 and PANoptosis necessitate additional verification. These constraints may affect the generalizability of conclusions and the depth of mechanistic interpretation. Subsequent studies should address these concerns through multicenter validation, algorithm enhancement, and empirical exploration.

## Conclusions

In conclusion, our comprehensive analysis of differentially expressed genes linked to PANoptosis in BLCA revealed two molecularly distinct subgroups with divergent prognostic outcomes, mutational profiles, and immune milieu features. This study clarifies BLCA progression through PANoptotic regulation, uncovering hitherto unrecognized pathogenic pathways. The established BLCA-Riskscore exhibits strong clinical value, indicating significant correlations with overall survival prognosis, response to immunotherapy, and vulnerability to molecularly targeted therapies. This classification technique enables the precise selection of patients for the most effective treatment options—either immunotherapy or targeted therapy. The primary gene PSMB5 significantly facilitates the progression of BLCA, and the control of PSMB5 by herbal drugs offers dual advantages for the treatment of BLCA and chemosensitization.

## Data Availability

The original contributions presented in the study are included in the article/**Supplementary Material**. Further inquiries can be directed to the corresponding authors.

## Supplementary material

Supplementary Figure 1 Comparison between risk score and clinical baseline measurements. **(A)** The risk score exhibits the greatest C-index. **(B)** The risk score has the highest AUC. **(C)** The DCA indicates that the risk score yields the most net benefit. **(D)** The standard curve indicates that the projected values are largely congruent with the actual values.

Supplementary Figure 2 29 significantly different drug susceptibility analyses between High-risk and Low-risk groups.

Supplementary Figure 3 Comparison between risk scores with clinical baseline measurements. The risk score exhibits the greatest utility when multiple machine learning techniques are employed to screen essential feature genes. **(A, B)** The feature importance boxplot indicates that the PSMB5 score is the most significant. Cyan signifies that this characteristic has been validated by the Boruta algorithm as a “confirmed” feature significantly correlated with the predictive variable. **(C)** SVM-RFE indicates that PSMB5 is nearest to the scatter points of SVM-RFE and possesses the highest average ranking. **(D)** The Lasso method indicates that the coefficient of PSMB5 possesses the most excellent absolute value. **(E, F)** The random forest analysis reveals that PSMB5 has the most excellent Mean Decrease Gini score.

Supplementary Figure 4 Overexpression of PSMB5 in *in vitro* experiments. **(A)** Overexpression of PSMB5 in J82 cells. **(B)** CCK-8 proliferation assay. **(C)** Colony formation assay. **(D)** Transwell migration assay in 24h. **(E)** Wound healing assay. **(F)** Flow cytometry analysis of cell apoptosis. All experimental techniques were conducted in three biological replicates with asterisk notation indicating non-significant (n.s.); **p* ≤ 0.05; ***p* ≤ 0.01; ****p *≤ 0.001; *****p *≤ 0.0001.

Supplementary Table 1 Three SNPs of PSMB5. SNP, single nucleotide polymorphism; SE, standard error; Effect Allele Frequency.

Supplementary Table 2 The result of MR between PSMB5 and BLCA. BLCA, bladder cancer;SE, standard error; OR, Odds Ratio; 95%CI, 95% Confidence Interval.
