# Supplementary figures and images for: Targeting PSMB5-induced PANoptosis in bladder cancer: multi-omics insights and TCM candidate discovery
Source: Front Immunol. 2025 Dec 2;16:1656682. doi: 10.3389/fimmu.2025.1656682 (PMC12705637; doi:10.3389/fimmu.2025.1656682)

Fig. 9 A

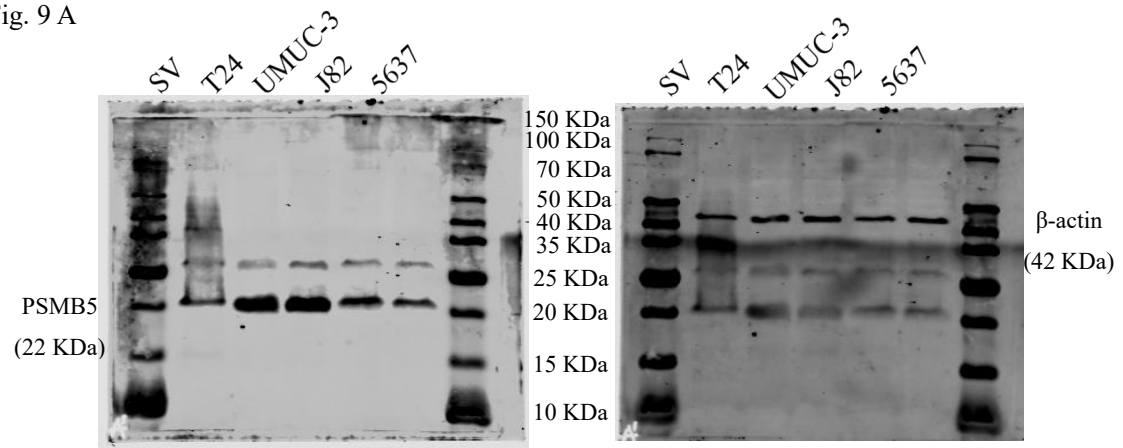

Fig. 9 B

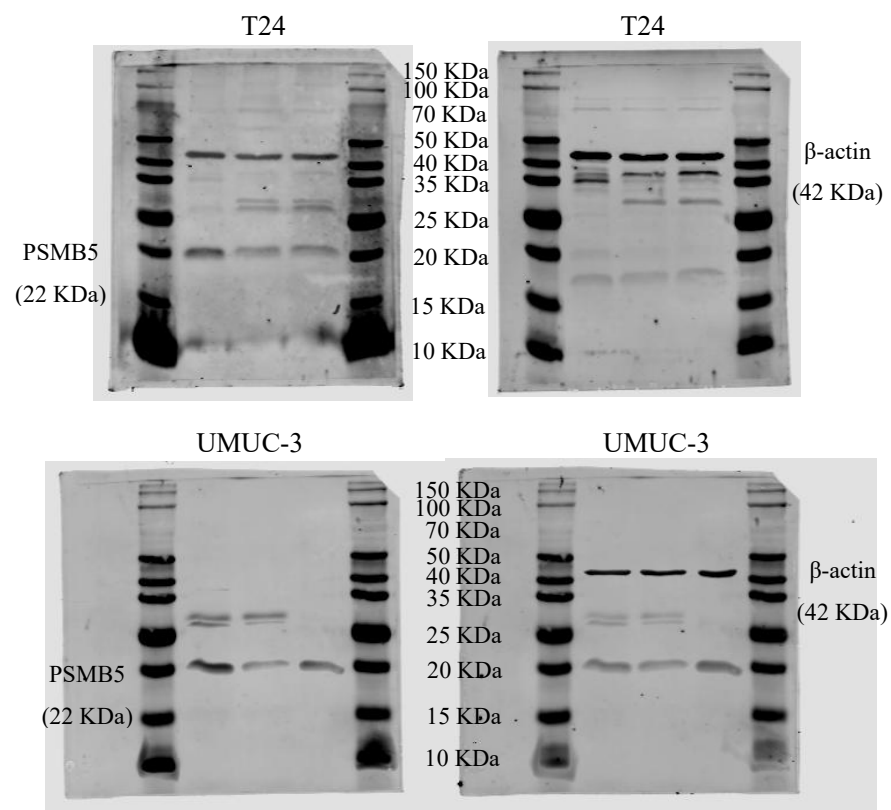

Supplement: Supplementary file 1 [file DataSheet1.pdf]

Supplementary Fig.4 A

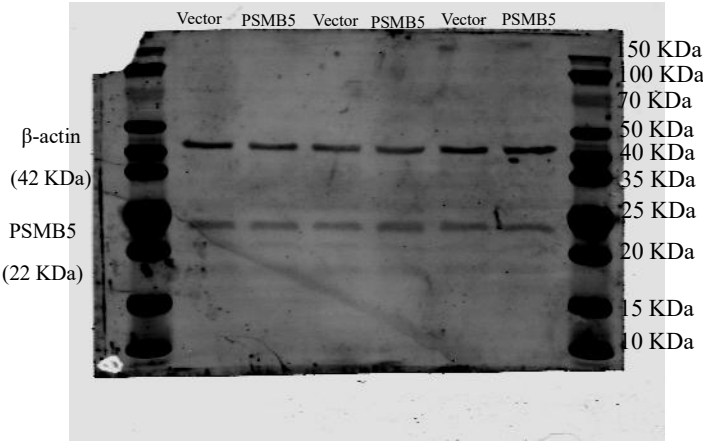

Supplement: Supplementary file 2 [file DataSheet2.pdf]

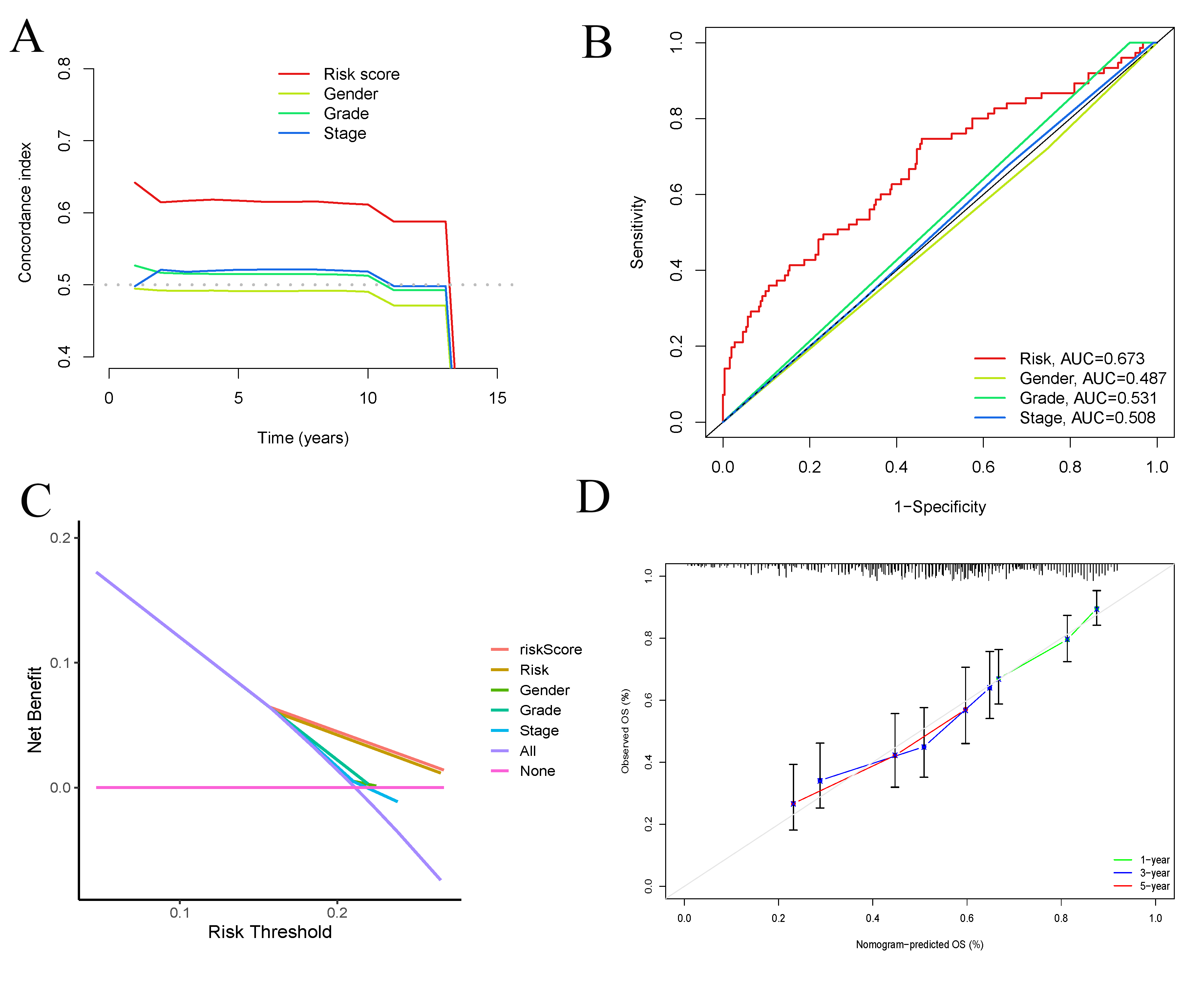

Supplement: Supplementary Figure 1 — Comparison between risk score and clinical baseline measurements. (A) The risk score exhibits the greatest C-index. (B) The risk score has the highest AUC. (C) The DCA indicates that the risk score yields the most net benefit. (D) The standard curve indicates that the projected values are largely congruent with the actual values. [file Image1.tif]

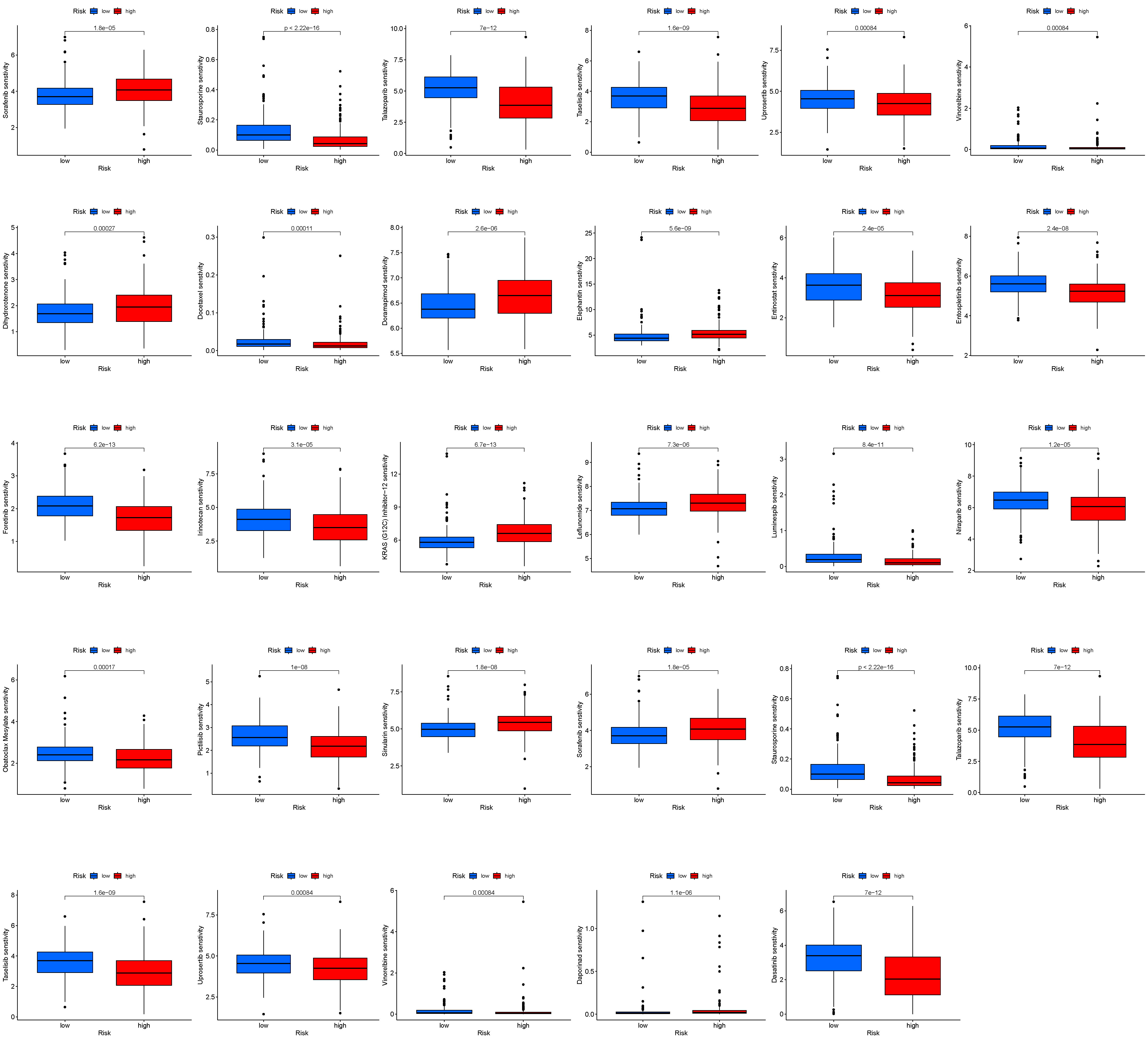

Supplement: Supplementary Figure 2 — 29 significantly different drug susceptibility analyses between High-risk and Low-risk groups. [file Image2.tif]

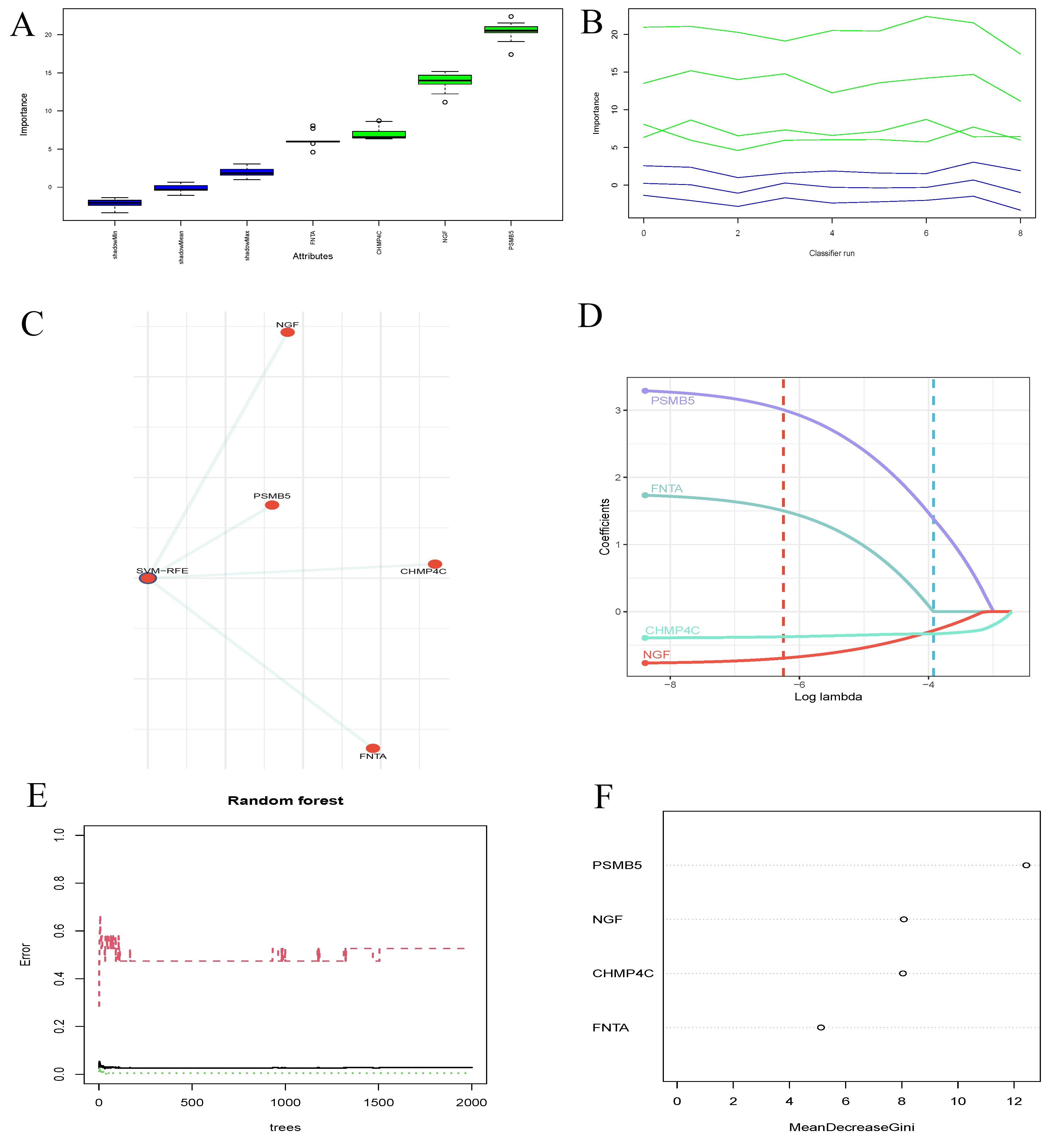

Supplement: Supplementary Figure 3 — Comparison between risk scores with clinical baseline measurements. The risk score exhibits the greatest utility when multiple machine learning techniques are employed to screen essential feature genes. (A, B) The feature importance boxplot indicates that the PSMB5 score is the most significant. Cyan signifies that this characteristic has been validated by the Boruta algorithm as a “confirmed” feature significantly correlated with the predictive variable. (C) SVM-RFE indicates that PSMB5 is nearest to the scatter points of SVM-RFE and possesses the highest average ranking. (D) The Lasso method indicates that the coefficient of PSMB5 possesses the most excellent absolute value. (E, F) The random forest analysis reveals that PSMB5 has the most excellent Mean Decrease Gini score. [file Image3.tif]

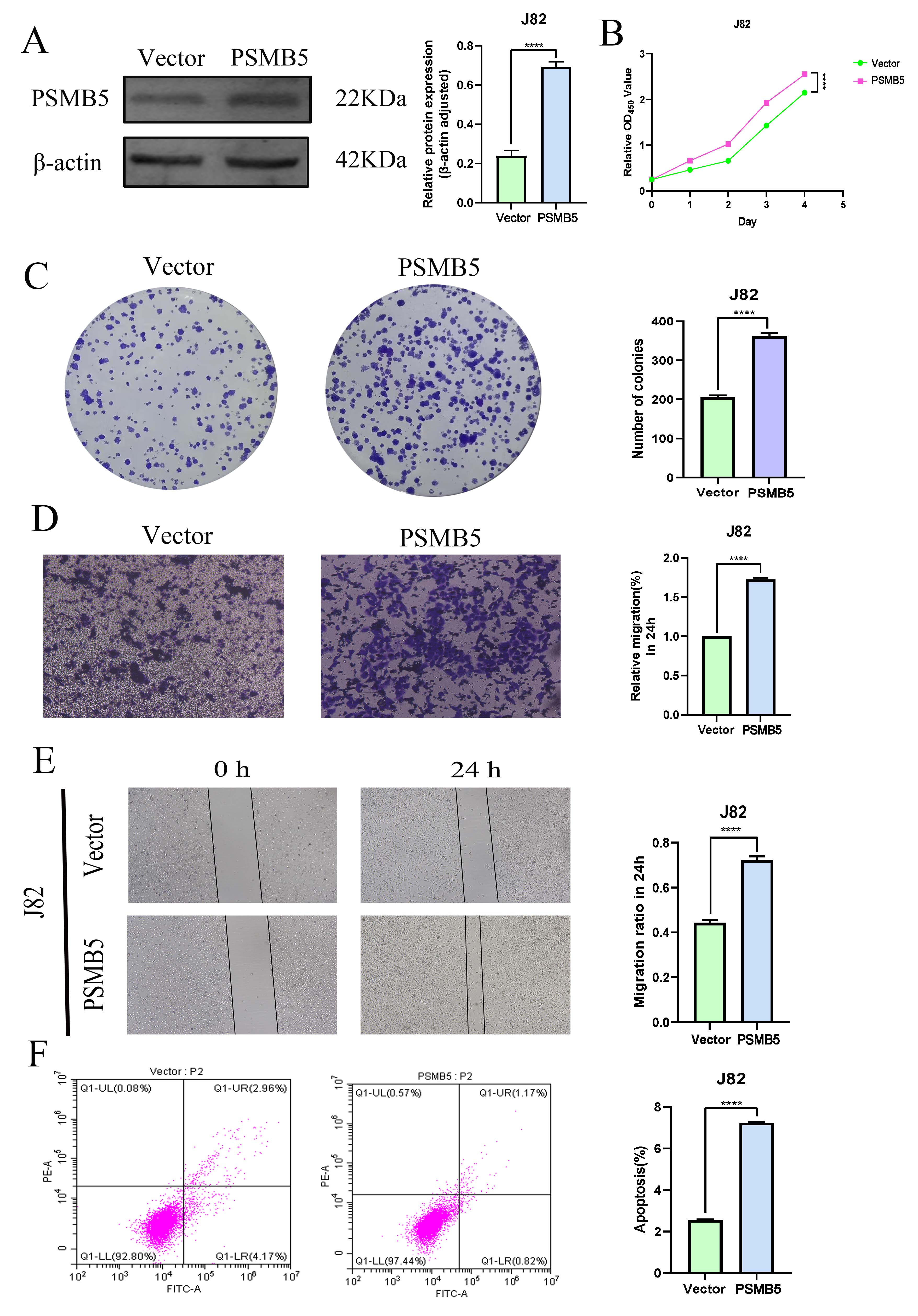

Supplement: Supplementary Figure 4 — Overexpression of PSMB5 in in vitro experiments. (A) Overexpression of PSMB5 in J82 cells. (B) CCK-8 proliferation assay. (C) Colony formation assay. (D) Transwell migration assay in 24h. (E) Wound healing assay. (F) Flow cytometry analysis of cell apoptosis. All experimental techniques were conducted in three biological replicates with asterisk notation indicating non-significant (n.s.); *p ≤ 0.05; **p ≤ 0.01; ***p ≤ 0.001; ****p ≤ 0.0001. [file Image4.jpeg]
