# Supplementary material for: Targeting PSMB5-induced PANoptosis in bladder cancer: multi-omics insights and TCM candidate discovery
Source: Front Immunol. 2025 Dec 2;16:1656682. doi: 10.3389/fimmu.2025.1656682 (PMC12705637; doi:10.3389/fimmu.2025.1656682)
Supplement: Supplementary Table 1 — Three SNPs of PSMB5. SNP, single nucleotide polymorphism; SE, standard error; Effect Allele Frequency. [file Table1.docx]

**Supplementary Table 1 Three SNPs of PSMB5.**

| Exposure | SNP | β | SE | EAF | *P*-value | R_2_ | F |
| --- | --- | --- | --- | --- | --- | --- | --- |
| PSMB5 | rs12590429 | 0.268 | 0.022 | 0.078 | 5.85e^-34^ | 0.005 | 147.574 |
| PSMB5 | rs117058979 | 0.288 | 0.045 | 0.018 | 2.04e^-10^ | 0.002 | 40.428 |
| PSMB5 | rs11543947 | -0.486 | 0.023 | 0.072 | 9.04e^-102^ | 0.016 | 458.704 |

SNP, single nucleotide polymorphism; SE, standard error; Effect Allele Frequency.

**Supplementary Table 2 The result of MR between PSMB5 and BLCA.**

| Outcome | Exposure | Method | β | SE | *P*-value | OR | OR 95%CI |
| --- | --- | --- | --- | --- | --- | --- | --- |
| BLCA | PSMB5 | Wald ratio | 0.818 | 0.414 | 0.048 | 2.267 | 1.008-5.097 |

BLCA, bladder cancer;SE, standard error; OR, Odds Ratio; 95%CI, 95% Confidence Interval.
